# Gut probiotic bacteria of *Barbonymus gonionotus* improve growth, hematological parameters and reproductive performances of the host

**DOI:** 10.1038/s41598-021-90158-x

**Published:** 2021-05-21

**Authors:** Mohammad Abdus Salam, Md. Ariful Islam, Sulav Indra Paul, Md. Mahbubur Rahman, Mohammad Lutfar Rahman, Fatama Islam, Ashikur Rahman, Dinesh Chandra Shaha, Md Shah Alam, Tofazzal Islam

**Affiliations:** 1 Department of Genetics and Fish Breeding, Faculty of Fisheries, Bangabandhu Sheikh Mujibur Rahman Agricultural University, Gazipur, 1706 Bangladesh; 2 Institute of Biotechnology and Genetic Engineering, Bangabandhu Sheikh Mujibur Rahman Agricultural University, Gazipur, 1706 Bangladesh; 3 Department of Fisheries Management, Faculty of Fisheries, Bangabandhu Sheikh Mujibur Rahman Agricultural University, Gazipur, 1706 Bangladesh; 4 Aquaculture Program Center for Marine Science, University of North Carolina Wilmington, 601, S. College Rd., Wilmington, NC 28403 USA

**Keywords:** Microbiology, Physiology

## Abstract

This study aimed to isolate and identify probiotic bacteria from the gut of *Barbonymus gonionotus* and evaluate their effects on growth, hematological parameters, and breeding performances of the host. Five probiotic bacteria viz*. Enterococcus xiangfangensis* (GFB-1), *Pseudomonas stutzeri* (GFB-2), *Bacillus subtilis* (GFB-3), *Citrobacter freundii* (GFB-4), and *P. aeruginosa* (GFB-5) were isolated and identified using *16S rRNA* gene sequencing. Application of a consortium of probiotic strains (1–3 × 1.35 × 10^9^ CFU kg^−1^) or individual strain such as GFB-1 (1.62 × 10^9^ CFU kg^−1^), GFB-2 (1.43 × 10^9^ CFU kg^−1^), GFB-3 (1.06 × 10^9^ CFU kg^−1^), GFB-4 (1.5 × 10^9^ CFU kg^−1^) or GFB-5 (1.43 × 10^9^ CFU kg^−1^feed) through feed significantly improved growth, histological and hematological parameters and reproductive performances of *B. gonionotus* compared to untreated control. Moreover, the application of these probiotics significantly increased gut lactic acid bacteria and activities of digestive enzymes but did not show any antibiotic resistance nor any cytotoxicity in vitro. The highest beneficial effects on treated fishes were recorded by the application of GFB-1, GFB-2, GFB-3, and a consortium of these bacteria (T2). This is the first report of the improvement of growth and health of *B. gonionotus* fishes by its gut bacteria.

## Introduction

The 2030 agenda for aquaculture is promoting sustainability by using natural resources as opposed to antibiotic prevention. Surprisingly, world fish production is highly increased to about 171 million tonnes in 2016, where aquaculture represented 47% of the total^1^. In Bangladesh, carp production is about 1.19 million metric tonnes, which is about 32.6% of the total fish production^2^. However, average fish production in aquaculture of Bangladesh is still much lower than many other carp producing countries like China. In this respect, a minor carp, *Barbonymus gonionotus* (Bleeker, 1850), commonly known as silver barb in aquaculture, widely distributed in almost all the countries in the world especially in South East Asian countries for aquaculture^3^. Because of its high popularity, its distribution has been widely extended by human introduction. It belongs to the Cyprinidae family which is a potential aquaculture candidate for enhancing fish production. This species is widely used in polyculture as well as weed control in South East Asia and also important fish species for integrated rice-fish farming^4^. The interest in *B. gonionotus* as a means of biological control of weeds instead of grass carp that destroy the plants^4^. It can thrive well in environmentally stressful conditions such as high stocking density^5^ and salinity^6^ coupled with its fast growth rate (mean 0.66 g per day)^6^ and taste that made it a popular culturable species^6^. It has the highest protein content (17.5%) among the freshwater fishes^7^ and more productivity (1976.21 kg ha^−1^/90 days)^6^ in commercial aquaculture. Overcrowding tends to more than 300 kg ha^−1^^6^ adversely affect the nonspecific immune system^8^ of fishes as well as fish become more susceptible to diseases especially motile *Aeromonas* septicemia^9^, streptococcosis^10^. Bacterial diseases hamper the colossal amount of fish production in aquaculture, and application of the chemicals such as antibiotics in health management is commonly practiced^11^. Antibiotics are applied as a traditional strategy to control bacterial fish diseases such as motile *Aeromonas* septicemia in freshwater and also for improving growth performances^12,13^. More than 70% of antibiotics are used in aquaculture operations wind up in the environment and plasmid carrying resistance genes can be transferred from fish pathogen to humane pathogens^13^. A few studies showed that probiotic bacteria increase immune response and growth performances in fish^14,15^. Several lines of evidence suggest that application of the native host developed probiotic strains of probiotic bacteria improve the health of host fishes by decreasing the mortality rate^16^.

In the last decades, many native hosts developed strains of probiotic bacteria that were isolated from the terrestrial hosts and applied in aquaculture without any verification of their effects on the physiology of fishes^17^. In many cases, those probiotic strains showed inconsistent results. It is reasonable to hypothesize that the probiotic bacteria isolated from the guts of native fishes could be the potential candidates for the promotion of sustainable aquaculture. Host-associated probiotics boosted the growth, immunity, and reproductive performances of the fish^17,18^. Recently, a total of twelve probiotic bacterial isolates collected from the digestive systems of three temperate fish species such as *Scophthalmus maximus, Platichthys flesus* and *Limanda limanda*^19^ and *Oreochromis niloticus* associated two probiotic bacterial strains *Lactobacillus plantarum* N11 and *Bacillus velezensis* H3.1^17^, found to adapt better than those derived from the terrestrial hosts. The probiotic bacteria can cause beneficial changes in morphology, the ratio of beneficial and pathogenic bacteria of the gut, or activity of intestinal microbiota, improving the absorption and digestion of nutrients, as well as cause improvements of the immune system, generating positive effects on host health^20,21^. Recently fish host-associated bacterial strains have been thoroughly examined for specific health effects of aquatic organisms. However, most authors generally consider growth, immunity, and reproductive performances^17,18^ without focus on the possibility of histological changes caused in the internal organs like the intestine and liver as a result of probiotic use. Probiotic bacterial strains enhanced the absorption area of the intestine through the increment of intestinal microvilli of fish^22^. The probiotic *L. plantarum* improves the liver tissue by reducing the lesion of the tissue of *O. niloticus*^23^. The fertilization, ovulation, hatching, and survival rate of fish larvae in the hatcheries are often not at the desirable level because of unexpected seed mortality^24^. Some hatcheries use commercial probiotic bacteria for reducing seed mortality^19^. However, the commercial probiotics used in aquaculture feed formulation are expensive and inconsistent in their efficacy^18^. In this aspect, genetically characterized probiotic bacteria isolated from the gut of native fishes could be used as cheap and eco-friendly agents in enhancing growth performance, hematological parameters, and breeding performances of fishes for promoting sustainable aquaculture. Therefore, the objectives of the present study were to (a) isolate and identify probiotic bacteria from the gut of *B. gonionotus* using *16S rRNA* gene sequencing; (b) investigate the effects of individual and consortium application of the identified probiotic bacteria on growth and hematological parameters; and (c) evaluate their influence on reproductive performances of the host fishes.

## Results

### Isolation and molecular identification of probiotic bacteria from the gut of *B. gonionotus*

**Figure 1:**
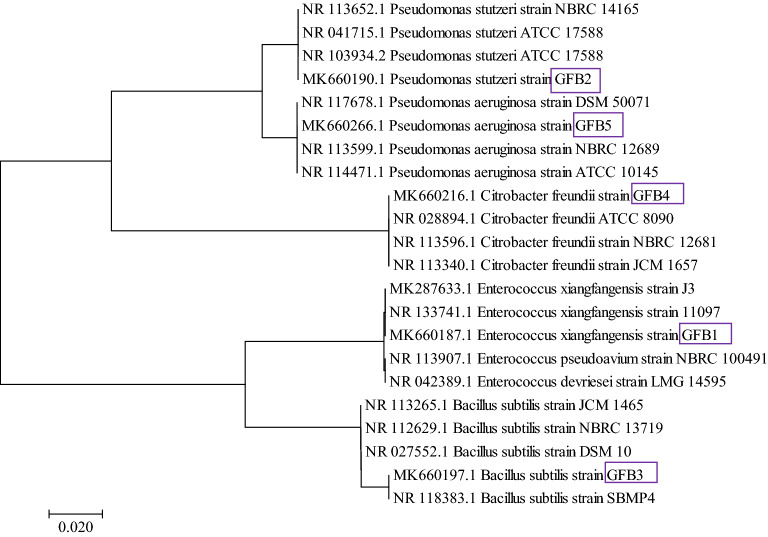
Phylogenetic relationship of strain GFB-1 (MK660187.1), GFB-2 (MK660190.1), GFB-3 (MK660197.1), GFB-4 (MK660216.1) and GFB-5 (MK660266.1) with their corresponding genus. The evolutionary distances were computed using the Maximum Composite Likelihood method. Evolutionary analyses were conducted in MEGA7.

Five morphologically distinct colonies of bacteria were isolated and purified from the gut of healthy individuals of *B. gonionotus*. Morphological studies of these isolates revealed that GFB-1 and GFB-(2-5) were cocci and rod-shaped, respectively. The colony characters, morphological features, physiological and biochemical properties of the five isolated probiotic bacteria are summarized in Table 1. The *16S rRNA* gene sequencing data of these isolates (GFB-1 to GFB-5) exhibited 99.93 to 100% homology with *Enterococcus xiangfangensis* (100%), *Pseudomonas stutzeri* (100%), *Bacillus subtilis* (99.93%), *Citrobacter freundii* (99.93%) and *P. aeruginosa* (100%), respectively. The sequences of these bacterial strains have been deposited in the NCBI GenBank. In the phylogenetic tree constructed using the neighbor-joining method, the strains GFB-1 (MK660187.1), GFB-2 (MK660190.1), GFB-3 (MK660197.1), GFB-4 (MK660216.1), and GFB-5 (MK660266.1) formed clusters with their corresponding species (Fig. 1).

**Table 1:** Morphological, physiological and biochemical characters of probiotic isolates collected from *B. gonionotus* gut.

Test type	Tests	Characteristics				
		GFB-1	GFB-2	GFB-3	GFB-4	GFB-5
Colony characters	Size	S	S	M	M	M
	Type	R	R	R	R	R
	Color	Whitish	Creamy	Whitish	Whitish	Whitish
	Shape	C	C	P	C	C
Morphological character	Shape	Cocci	Rod	Rod	Rod	Rod
Physiological characters	Motility	−	+	+	+	+
	Growth at 10 °C	+	+	+	+	+
	Growth at 45 °C	+	+	+	+	+
	Growth in 6.5% NaCl	+	+	+	+	+
Biochemical characters	Gram’s staining	+	−	+	−	−
	Catalase	−	+	+	+	+
	Oxidase	−	+	−	−	−
	Oxidative-Fermentative	F	F	O	F	O
	Methyl Red	−	−	−	+	−
	Voges-Proskauer	+	−	+	−	−
	Indole	−	−	−	−	−

S, small; M, medium; R, round; C, convex; P, Plain; O, oxidative; F, fermentative; +, positive; −, negative.

### Bacterial viability in the formulated feed

In order to confirm the bacterial viability in the formulated feeds, we counted the total viable bacteria in formulated feed weekly. Interestingly, all the five probiotic preparation were characterized with optimum level of viable bacterial cells (GFB-1 = 1.31 × 10^9^ CFU ml^−1^; GFB-2 = 1.18 × 10^9^ CFU ml^−1^; GFB-3 = 1.12 × 10^9^ CFU ml^−1^; GFB-4 = 1.36 × 10^9^ CFU ml^−1^ and GFB-5 = 1.19 × 10^9^ CFU ml^−1^). Then, the five probiotic preparation at the dose of 50 ml kg^−1^ of feed offered the possibility of preparing feeds with viable cell concentrations of GFB-1 = 1.62 × 10^9^ CFU kg^−1^ feed; GFB-2 = 1.43 × 10^9^ CFU kg^−1^ feed; GFB-3 = 1.06 × 10^9^ CFU kg^−1^ feed; GFB-4 = 1.5 × 10^9^ kg^−1^ feed and GFB-5 = 1.13 × 10^9^ kg^−1^ feed. All the five concentrations of probiotic viable cells in feeds were similar to the recommended dose (10^9^ CFU kg^−1^ feed)^25–27^.

### Enhancement of growth of *B. gonionotus* by probiotic bacteria

**Figure 2:**
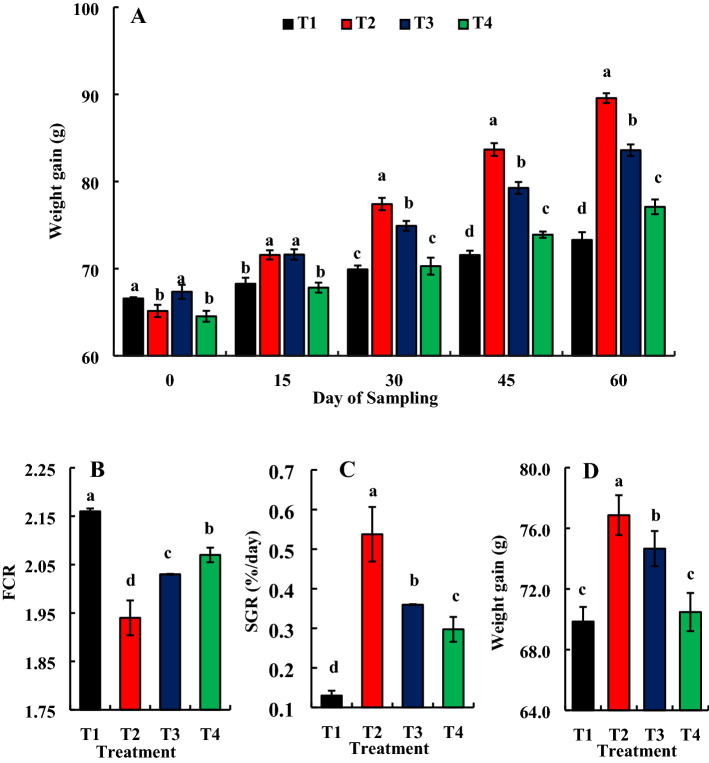
Assessment of consortium effects of five probiotic bacterial strains viz*.* GFB-1, GFB-2, GFB-3, GFB-4 and GFB-5 isolated from the gut of *B. gonionotus* on growth parameters (A, B, C and D) of the host fishes. Five probiotic bacterial strains were mixed with 1:1:1:1:1 ratio before adding in the formulated feed and fishes were reared for 60 days. Here, T1 = 0 CFU kg^−1^ feed (control); T2 = 1.35 × 10^9^ CFU kg^−1^ feed; T3 = 2 × 1.35 × 10^9^ CFU kg^−1^ feed and T4 = 3 × 1.35 × 10^9^ CFU kg^−1^ feed. One way ANOVA was performed for analyzing the data of three replicated experiment and data in column varies significantly in LSD at *P* < 0.05 (Statistix 10). Different letter bars indicate significant variations in (A) weight gain, (B) food conversion ratio (FCR) and (C) specific growth rate (SGR) of the host fishes in different groups by the isolates at *P* < 0.05 (Statistix 10). Error bar =  ± SD; n = 48. **(**D**)** Weight gain data collected were repeated statistically analysed using ANOVA to test significance results (*P* < 0.05) between means. Standard error (± SE) was calculate to identify the range of means. These statistical analyses were performed with the aid of the computer software SPSS 26.0 version. R^2^ values are as follows: T1 (Control) (0.801); T2 (0.834); T3 (0.807); T4 (0.799).

**Figure 3:**
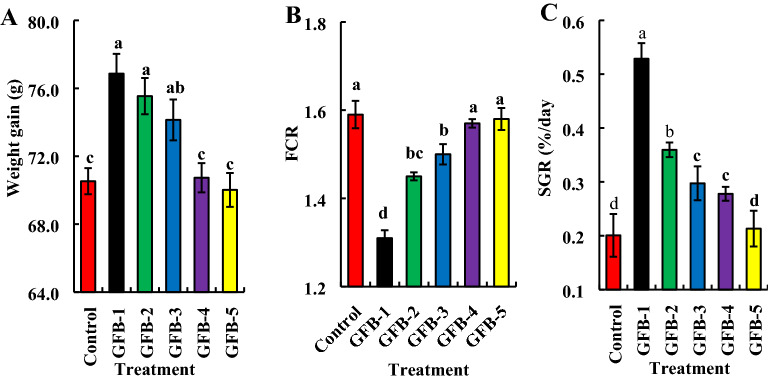
Assessment of individual effects of five probiotic bacterial strains viz. GFB-1, GFB-2, GFB-3, GFB-4 and GFB-5 isolated from the gut of *B. gonionotus* on growth parameters (A, B, and C) of the host fishes. One way ANOVA was performed for analyzing the data of three replicated experiment and data in column varies significantly in LSD at *P* < 0.05 (Statistix 10). Different letter bars indicate significant variations in (**A**) Weight gain data collected were repeated statistically analysed using ANOVA to test significance results (*P* < 0.05) between means, (B) food conversion ratio (FCR) and (C) specific growth rate (SGR) of fish in different groups by the isolates at *P* < 0.05 (Statistix 10). Error bar =  ± SD; n = 48. Standard error (± SE) was calculate to identify the range of means. These statistical analyses were performed with the aid of the computer software SPSS 26.0 version. R^2^ values are as follows: Control (0.747); GFB-1 (0.865); GFB-2 (0.838); GFB-3 (0.846); GFB-4 (0.748); GFB-5 (0.687).

To evaluate the consortium effect of the newly isolated probiotic bacteria on the growth of *B. gonionotus*, we fed fishes bacteria supplemented formulated feed with a consortium of five probiotic bacteria at the ratio of 1:1:1:1:1 (1.35 × 10^9^ to 3 × 1.35 × 10^9^ CFU kg^−1^ feed), and data were recorded at day 0, 15, 30, 45 and 60 after the treatment (Fig. 2). Another experiment was performed feeding fishes with the individual strain of bacteria with the formulated feed (Fig. 3). In the case of a consortium application, the weight gains of fishes were 6.72 ± 0.68, 24.43 ± 1.12, 16.25 ± 0.84, and 12.56 ± 0.47 g in T1, T2, T3, and T4, respectively after the end of 60 days (Fig. 2A). The highest body weight gain was found in fishes fed feed supplemented with bacteria at the dose of 1.35 × 10^9^ CFU kg^−1^ feed when data were recorded at day 45 and 60 days after the treatment (Fig. 2A). Significantly (*P* < 0.05) higher body weight gain was recorded in all probiotic bacteria treated fishes compared to the untreated control fishes at both 45 and 60 days after the treatment (Fig. 2A). Interestingly, a decreasing trend of body weight gain was found in the treatment of fishes with higher doses of probiotic bacteria than at 1.35 × 10^9^ CFU kg^−1^ feed (Fig. 2A). Repeated statistical analyses also showed a similar trend that significantly the highest weight gain was found in fishes fed feed supplemented with bacteria at the dose of T2 (1.35 × 10^9^ CFU kg^−1^ feed) when data were recorded 60 days after treatment (Fig. 2D). Surprisingly, a decreasing trend of body weight gain was found in the treatment of fishes with higher doses of probiotic bacteria than at 1.35 × 10^9^ CFU kg^−1^ feed (Fig. 2D). A similar trend of effects of probiotic bacteria was also found in the specific growth rate (SGR%/day) of fishes (Fig. 2C). The food conversion ratio (FCR) in T1, T2, T3, and T4 were 2.17 ± 0.006, 1.94 ± 0.036, 2.03 ± 0.001, and 2.07 ± 0.015, respectively after 60 days (Fig. 2B). A reverse trend of results was obtained in the case of FCR (Fig. 2B). The highest FCR was recorded in the fish treated with no probiotic bacteria. On the other hand, the FCR was significantly increased with the increasing doses of the probiotic bacteria (Fig. 2B).

Almost similar results were found in the case of fishes fed feed supplemented with individual bacteria (Fig. 3). Repeated statistical analyses showed significantly (*P* < 0.05) the highest weight gain was found in GFB-1 (1.62 × 10^9^ CFU kg^−1^ feed) when data were recorded 60 days after treatment (Fig. 3A). Amusingly, a decreasing trend of body weight gain was found in the treatment of fishes with higher doses of probiotic bacteria than at 1.62 × 10^9^ CFU kg^−1^ feed (Fig. 3A). The highest specific growth rate (%/day) (SGR) 0.516 ± 0.029 was found in GFB-1 and the lowest SGR 0.303 ± 0.026 was found in GFB-4 (Fig. 3C). Among the isolates, treatment with GFB-1 showed the significantly highest body weight gain (g) and percent of SGR followed by GFB-2 and GFB-3 (Fig. 3A, C). The GFB-5 induced the lowest body weight gain (g) of treated fishes compared to any other individual probiotic bacterial treatment. However, the FCRs were significantly (*P* < 0.05) higher in fishes treated with GFB-4, GFB-5, and no probiotic bacteria (untreated control). The lowest FCR was recorded in fishes fed feed supplemented with GFB-1 followed by GFB-2 and GFB-3 (Fig. 3B).

**Figure 4:**
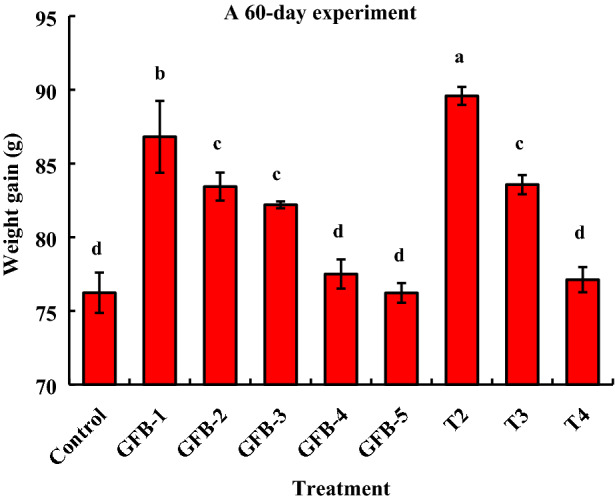
Comparative study between assessment of individual effects of five probiotic bacterial strains viz. GFB-1, GFB-2, GFB-3, GFB-4 and GFB-5 and consortium effects of five bacterial strains viz T2, T3 and T4 isolated from the gut of *B. gonionotus* on weight gain of the host fish. One way ANOVA was performed for analyzing the data of three replicated experiment and data in column varies significantly in LSD at *P* < 0.05 (Statistix 10). Different letter bars indicate significant variations in weight gain of fish in different groups by the isolates at *P* < 0.05 (Statistix 10). Error bar =  ± SD; n = 48.

Significantly (*P* < 0.05) the highest weight gain was found in fishes fed feed supplemented with a consortium of all five bacterial isolates at the dose of 1.35 × 10^9^ CFU kg^−1^ feed compared to the fishes fed feed supplemented with GFB-1, GFB-2, or GFB-3 (Fig. 4).

### Probiotic treated feeds enhance the length of villi of intestine and improve liver cells of *B. gonionotus*

**Figure 5:**
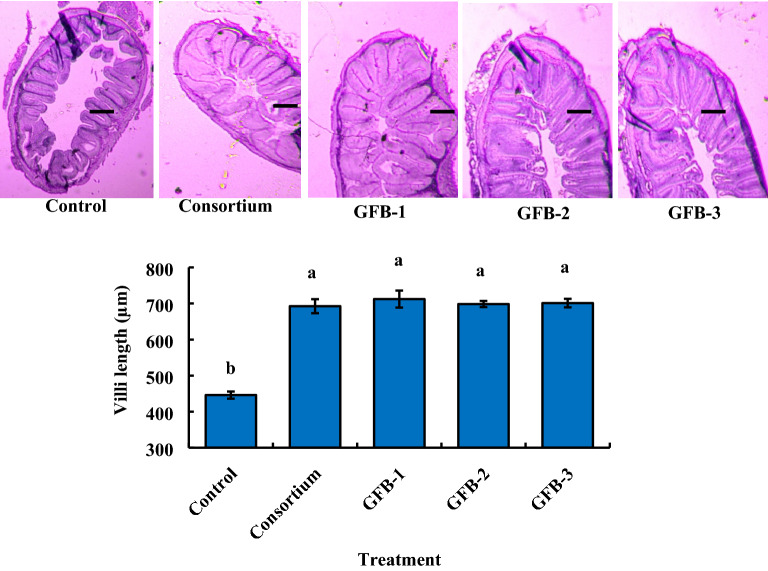
Histological analysis of the effects of probiotic bacteria supplementation on villi length of intestine of *B. gonionotus* (A-F). Fishes were fed on (A) control diet (0 CFU kg^−1^ feed), (B) consortium (T2) (1.35 × 10^9^ CFU kg^−1^ feed), (C) GFB-1 (1.62 × 10^9^ CFU kg^−1^ feed), (D) GFB-2 (1.43 × 10^9^ CFU kg^−1^ feed) and (E) GFB-3 (1.06 × 10^9^ CFU kg^−1^ feed). (F) It shows probiotic bacteria treated intestinal villi length compared to control (n = 6). Different letter bars indicate significant variations in intestinal villi length of fishes (F) fishes in different groups by the isolates at *P* < 0.05 (Statistix 10). Scale bar = 100 µm; image 4x; H & E 200. R^2^ values are as follows: Control (0.737), Consortium (0.639), GFB-1 (0.946). GFB-2 (0.746), and GFB-3 (0.825).

**Figure 6:**
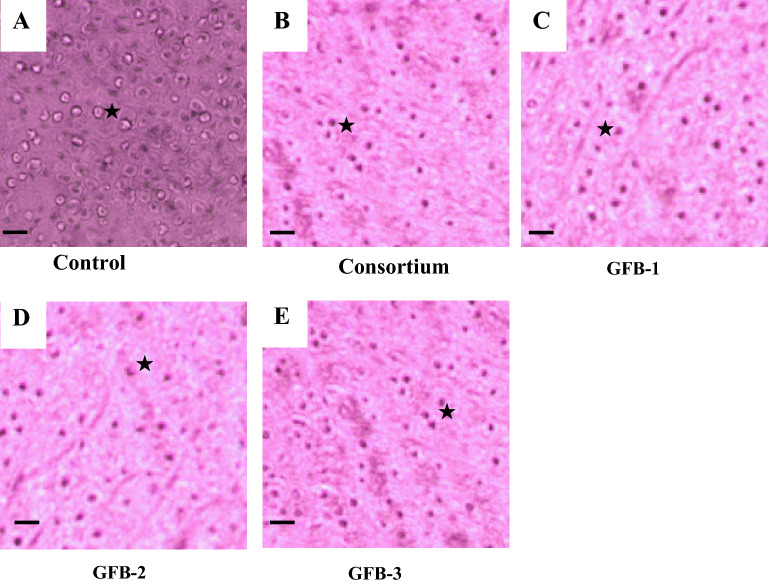
Histological analysis of the effects of probiotic supplementation on liver (A-E) of *B. gonionotus*. Fishes were fed on (A) control diet (0 CFU kg^−1^ feed), (B) consortium (T2) (1.35 × 10^9^ CFU kg^−1^ feed), (C) GFB-1 (1.62 × 10^9^ CFU kg^−1^ feed), (D) GFB-2 (1.43 × 10^9^ CFU kg^−1^ feed) and (E) GFB-3 (1.06 × 10^9^ CFU kg^−1^ feed). (A) It shows unclear hepatocyte cells (arrow) with irregular shaped nucleus (asterisk). (B), (C), (D) and (E) show the hepatocytes with regular shaped nucleus. Six fishes were considered for each treatment (n = 6). Image 40x; scale bar = 50 µm; H & E 200.

After 60 days of probiotics treatment, histological analyses of the intestine were done to determine the effects of both consortium and individual probiotic treatments on the intestinal structures of fishes. Interestingly, the histological study showed that application of both consortium (1.35 × 10^9^ CFU kg^−1^ feed) and individual bacterial probiotic strains, GFB-1 (1.62 × 10^9^ CFU kg^−1^ feed), GFB-2 (1.43 × 10^9^ CFU kg^−1^ feed) and GFB-3 (1.06 × 10^9^ CFU kg^−1^ feed) significantly (*P* < 0.05) increased the length of villi of the intestine of fishes compared to the untreated control (Fig. 5). The highest length of intestinal microvilli was 712.317 ± 23.66 µm in GFB-1 and the lowest length of intestinal microvilli was 446.00 ± 9.85 µm in the untreated control (Fig. 5). In addition, both consortium and individual probiotic bacterial treatments (GFB-1, GFB-2, and GFB-3) also significantly (*P* < 0.05) improved the cells of the liver compared to untreated control (Fig. 6).

### Probiotic bacteria improve the hematological parameters of *B. gonionotus*

**Figure 7:**
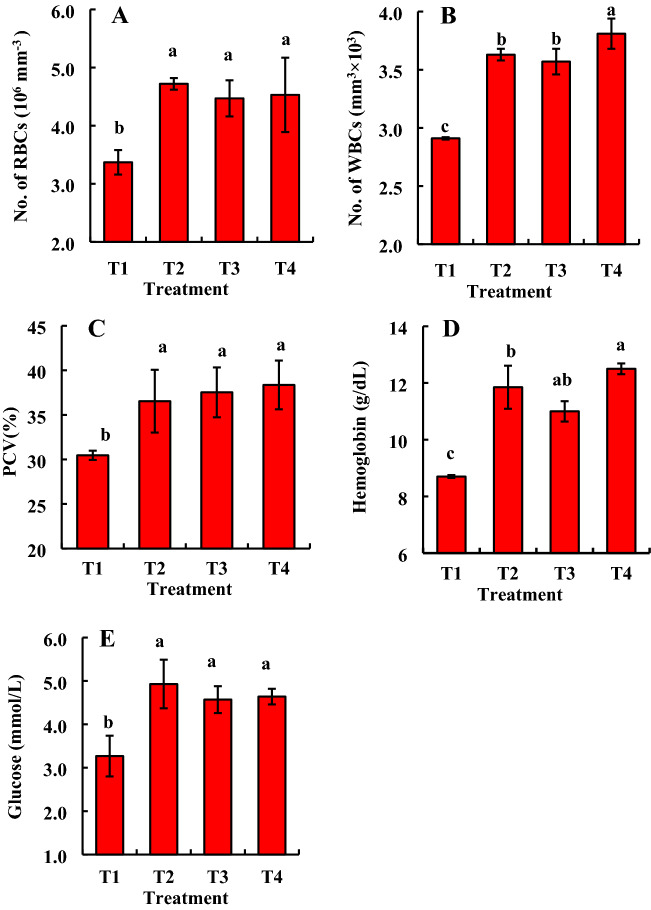
Assessment of consortium effects of bacterial probiotic strains viz*.* GFB-1, GFB-2, GFB-3, GFB-4 and GFB-5 isolated from the gut of *B. gonionotus* on hematological parameters (A, B, C, D and E) of the host fish. Blood was collected from nine fishes of each replication and 27 fishes of each treatment for estimating (A) red blood cell (RBC), (B) white blood cell (WBC), (C) packed cell volume (PCV), (D) hemoglobin level and (E) glucose level. One way ANOVA was performed for analyzing the data of three replicated experiment and data in column varies significantly in LSD at *P* < 0.05 (Statistix 10). Different letter bars indicate significant variations in RBC, WBC, PCV (%), hemoglobin and glucose level of the host fishes in different groups by the isolates at *P* < 0.05 (Statistix 10). Error bar =  ± SD; n = 27. R^2^ values are as follows: RBC = T1 (Control) (0.642), T2 (0.671), T3 (0.642), and T4 (0.997); WBC = T1 (Control) (0.712), T2 (0.738), T3 (0.976), and T4 (0.783); PCV = T1 (Control) (0.846), T2 (0.630), T3 (0.750), and T4 (0.629); Hemoglobin = T1 (Control) (0.977), T2 (0.713), T3 (0.929), and T4 (0.789); Glucose = T1 (Control) (0.803), T2 (0.635), T3 (0.724), and T4 (0.956).

**Figure 8:**
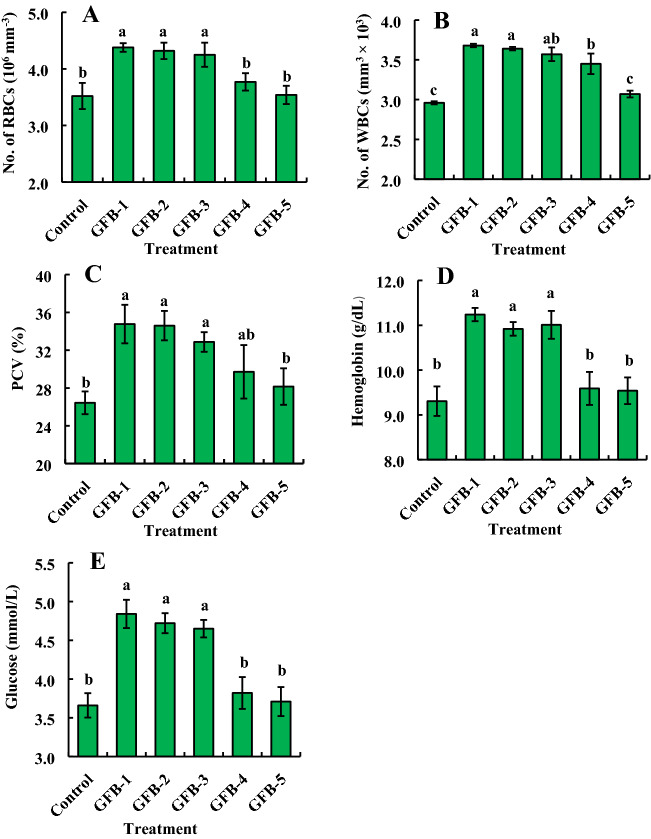
Assessment of individual effects of five isolated probiotic strains viz*.* GFB-1, GFB-2, GFB-3, GFB-4 and GFB-5 from the gut of *B. gonionotus* on hematological parameters (A, B, C, D and E) of the host fish. Blood was collected from ten fishes of each replication for estimating red blood cell (RBC), white blood cell (WBC), packed cell volume (PCV), hemoglobin level and glucose level. One way ANOVA was performed for analyzing the data of three replicated experiment and data in column varies significantly in LSD at *P* < 0.05 (Statistix 10). Different letter bars indicate significant variations in RBC, WBC, PCV (%), hemoglobin and glucose of the host fish in different groups by the isolates at *P* < 0.05 (Statistix 10). Error bar =  ± SD; n = 27. R^2^ values are as follows: RBC = Control (0.848), GFB-1 (0.921), GFB-2 (0.999), GFB-3 (0.993), GFB-4 (0.971), and GFB-5 (0.969); WBC = Control (0.912), GFB-1 (0.824), GFB-2 (0.892), GFB-3 (0.692), GFB-4 (0.997), and GFB-5 (0.951); PCV = Control (0.745), GFB-1 (0.713), GFB-2 (0.623), GFB-3 (0.822), GFB-4 (0.907), and GFB-5 (0.644); Hemoglobin = Control (0.978), GFB-1 (0.714), GFB-2 (0.644), GFB-3 (0.979), GFB-4 (0.678), and GFB-5 (0.749); Glucose = Control (0.957), GFB-1 (0.714), GFB-2 (0.669), GFB-3 (0.956), GFB-4 (0.520), and GFB-5 (0.607).

In order to evaluate the effects of the newly isolated probiotic bacteria on hematological parameters of *B. gonionotus*, we fed fishes bacteria supplemented formulated feed with the consortium of five probiotic bacteria at the ratio of 1:1:1:1:1 (1.35 × 10^9^ to 3 × 1.35 × 10^9^ CFU kg^−1^ feed) and the data were recorded at 60 days after treatment (Fig. 7). Another experiment was performed feeding fishes with the individual strain of bacteria with formulated feed (Fig. 8). In the case of a consortium application, RBC, PCV, and glucose level were significantly (*P* < 0.05) higher in fishes fed feed supplemented with bacteria at the dose of 1.35 × 10^9^ CFU kg^−1^ feed than fishes fed feed without probiotic bacteria (Fig. 7A, C, E) when data were recorded at day 60 after the treatment. On the other hand, WBC and hemoglobin level were found significantly (*P* < 0.05) higher in fishes fed feed supplemented with bacteria at the dose of 3 × 1.35 × 10^9^ CFU kg^−1^ feed than fishes fed feed without probiotic bacteria (Fig. 7B, D).

Almost similar results were found in the case of fishes fed feed supplemented with individual bacteria (Fig. 8). Treatments with all probiotic bacterial isolates, GFB-1, GFB-2, and GFB-3 significantly (*P* < 0.05) enhanced RBC of fishes compared to untreated control (Fig. 8A). Among the isolates, treatment with GFB-1 and GFB-2 gave the significantly highest WBC of *B. gonionotus* followed by GFB-3 and GFB-4 (Fig. 8B). The GFB-5 induced the lowest WBC compared to any other individual probiotic bacterial treatment (Fig. 8B). Interestingly, treatment with all probiotic bacterial isolates, GFB-1, GFB-2, and GFB-3 significantly (*P* < 0.05) enhanced PCV (%) and hemoglobin level of *B. gonionotus* compared to untreated control as well as GFB-4 and GFB-5 (Fig. 8C, D). Treatment with probiotic bacterial isolates, GFB-1, GFB-2, and GFB-3 significantly (*P* < 0.05) enhanced glucose level of *B. gonionotus* compared to untreated control and GFB-4 and GFB-5 (Fig. 8E).

**Figure 9:**
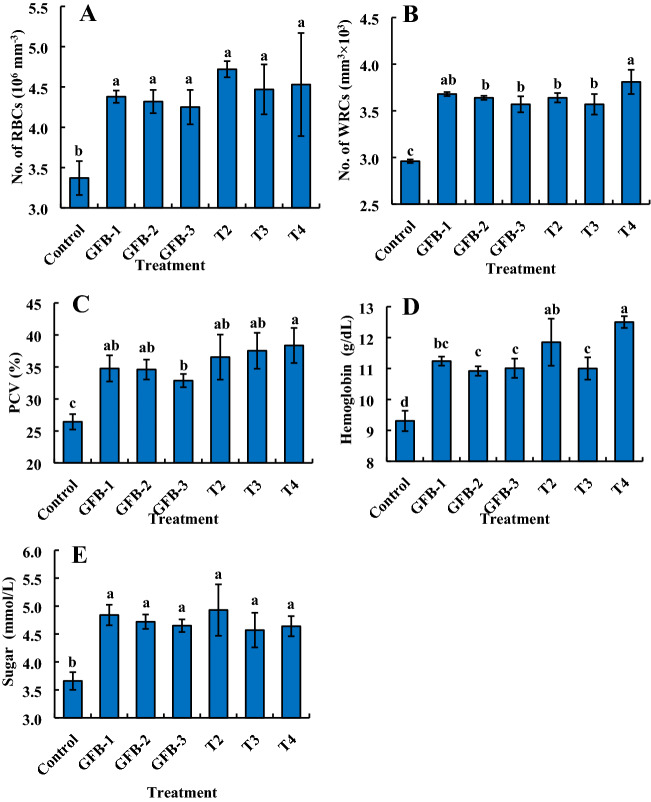
Comparative study between assessment of individual effects of five probiotic bacterial strains viz. GFB-1, GFB-2, GFB-3, GFB-4 and GFB-5 and consortium effects of five bacterial strains viz T2, T3 and T4 isolated from the gut of *B. gonionotus* on hematological parameters (A, B, C, D and E) of the host fish. One way ANOVA was performed for analyzing the data of three replicated experiment and data in column varies significantly in LSD at *P* < 0.05 (Statistix 10). Different letter bars indicate significant variations in RBC, WBC, PCV (%), hemoglobin and glucose of the host fish in different groups by the isolates at *P* < 0.05 (Statistix 10). Error bar =  ± SD; n = 27.

Significantly (*P* < 0.05) the highest number of RBCs and glucose level were found in fishes fed feed supplemented with the consortium of all five bacterial isolates at the dose of (1–3) 1.35 × 10^9^ CFU kg^−1^ feed and GFB-1, GFB-2, and GFB-3 compared to the fishes fed feed supplemented without probiotics (Fig. 9A, E). Interestingly, significantly (*P* < 0.05) the highest number of WBCs, PCV, and hemoglobin level were found in fishes fed feed supplemented with a consortium of all five bacterial isolates at the highest dose of 3 × 1.35 × 10^9^ CFU kg^−1^ feed compared to the fishes fed feed supplemented with T2, T3, GFB-1, GFB-2 or GFB-3 (Fig. 9B–D).

### Probiotic bacteria enhance the reproductive performances of the host fish

**Figure 10:**
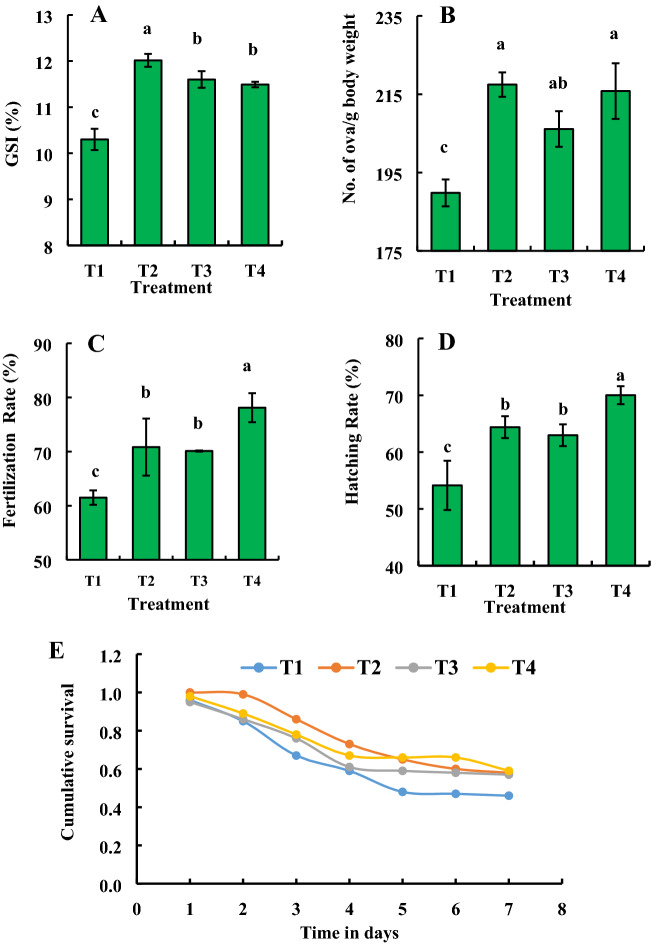
Assessment of consortium effects of probiotic bacterial strains viz*.* GFB-1, GFB-2, GFB-3, GFB-4 and GFB-5 isolated from the gut of *B. gonionotus* on reproductive performances (A, B, C, D and E) of the host fish. The fishes were stoked at 16 each replication at ratio of 1:3 of male and female for assessing (A) gonadosomatic index (GSI), (B) no. of ova/g body weight, (C) fertilization rate, (D) hatching rate and (E) cumulative survival of larva with a with a 95% CI (n = 100). One way ANOVA was performed for analyzing the data of three replicated experiment and data in column varies significantly in LSD at *P* < 0.05 (Statistix 10). Different letter bars indicate significant variations in GSI, no. of ova/ g body weight, fertilization rate, hatching rate and survival rate of hatchlings of the host fish by the isolates at *P* < 0.05 (Statistix 10). Error bar =  ± SD; n = 24.

**Figure 11:**
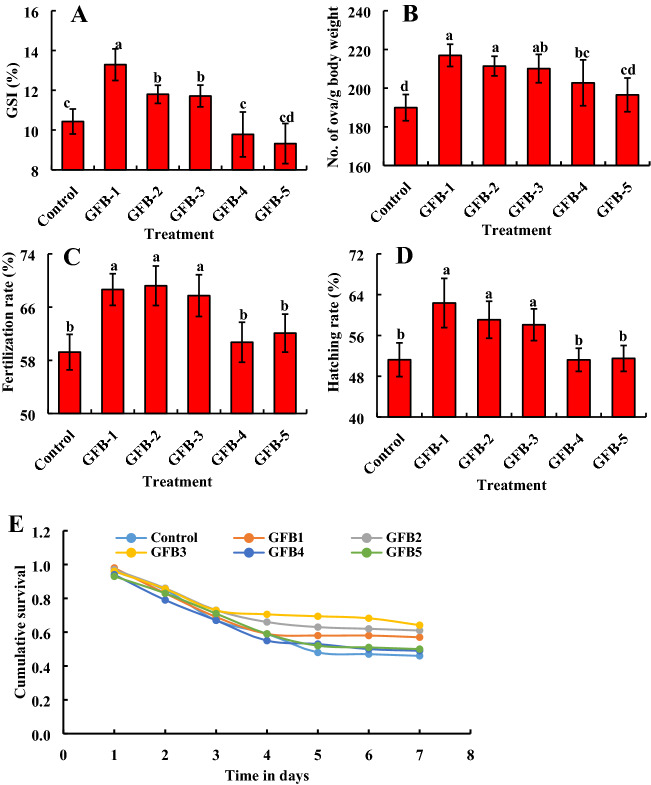
Assessment of individual of probiotic bacterial strains viz*.* GFB-1, GFB-2, GFB-3, GFB-4 and GFB-5 isolated from the gut of *B. gonionotus* on reproductive performances (A, B, C, D and E) of the host fish. The fishes were stoked at 16 each replication at ratio of 1:3 of male and female for assessing (**A**) gonadosomatic index (GSI), (B) no. of ova/g body weight, (C) fertilization rate, (D) hatching rate and (E) cumulative survival of larva with a with a 95% CI (n = 100). One way ANOVA was performed for analyzing the data of three replicated experiment and data in column varies significantly in LSD at *P* < 0.05 (Statistix 10). Different letter bars indicate significant variations in GSI, no. of ova/ g body weight, fertilization rate, hatching rate and survival rate of hatchlings of the host fish in different groups by the isolates at *P* < 0.05 (Statistix 10). Error bar =  ± SD; n = 24.

In order to assess the consortium effects of newly probiotic strains isolated from the gut of *B. gonionotus* on the reproductive performances of the host fishes, we selected a total of 96 sexually matured fishes for evaluating their breeding performances. We maintained the male and female ratio of 1:3. We fed fishes probiotic bacteria supplemented formulated feed with a consortium of five probiotic bacteria at the ratio of 1:1:1:1:1 (1.35 × 10^9^–3 × 1.35 × 10^9^ CFU kg^−1^ feed) and data were recorded at 60 days after the treatment (Fig. 10). We also selected a total of 144 sexually matured fishes at the male and female ratio of 1:3 for treatment with individual isolates, GFB-1, GFB-2, GFB-3, GFB-4, and GFB-5 (Fig. 11). In the case of a consortium application, the GSI and number of ova/g body weight were found significantly (*P* < 0.05) higher in fishes fed feed supplemented with probiotic bacteria at the dose of 1.35 × 10^9^ CFU kg^−1^ feed than fishes fed feed without probiotic bacteria (Fig. 10A, B). On the other hand, fertilization rate and hatching rate were found significantly (*P* < 0.05) higher in fishes fed feed supplemented with probiotic bacteria at the dose of 3 × 1.35 × 10^9^ CFU kg^−1^ feed than fishes fed feed without probiotic bacteria (Fig. 10C, D). The Kaplan–Meier survival analysis indicated that T4 treated larva exhibited the highest cumulative survival followed by T2 and T3 as well as control (Fig. 10E). Almost similar results were found in breeding parameters in the case of fishes fed feed supplemented with individual bacteria (Fig. 11). Treatments with all probiotic bacterial isolates such as GFB-1, GFB-2 and GFB-3 significantly (*P* < 0.05) enhanced GSI and number of ova/g body weight of *B. gonionotus* compared to untreated control (Fig. 11A, B). Moreover, GFB-1, GFB-2, and GFB-3 also significantly (*P* < 0.05) enhanced GSI and number of ova/g body weight of *B. gonionotus* compared to GFB-4 and GFB-5. Interestingly, the bacterial isolate GFB-1 significantly (*P* < 0.05) enhanced the GSI of *B. gonionotus* followed by GFB-4 and GFB-5 (Fig. 11A). Among the isolates, treatment with GFB-1, GFB-2, and GFB-3 showed a significantly (*P* < 0.05) higher rate of fertilization and hatching rate of *B. gonionotus* compared to untreated control as well as GFB-4 and GFB-5 (Fig. 11C, D). Moreover, the Kaplan–Meier survival analysis indicated that GFB-3 treated larva exhibited the highest cumulative survival followed by GFB-2, GFB-1, GFB-5 and GFB-4 (Fig. 11E).

**Figure 12:**
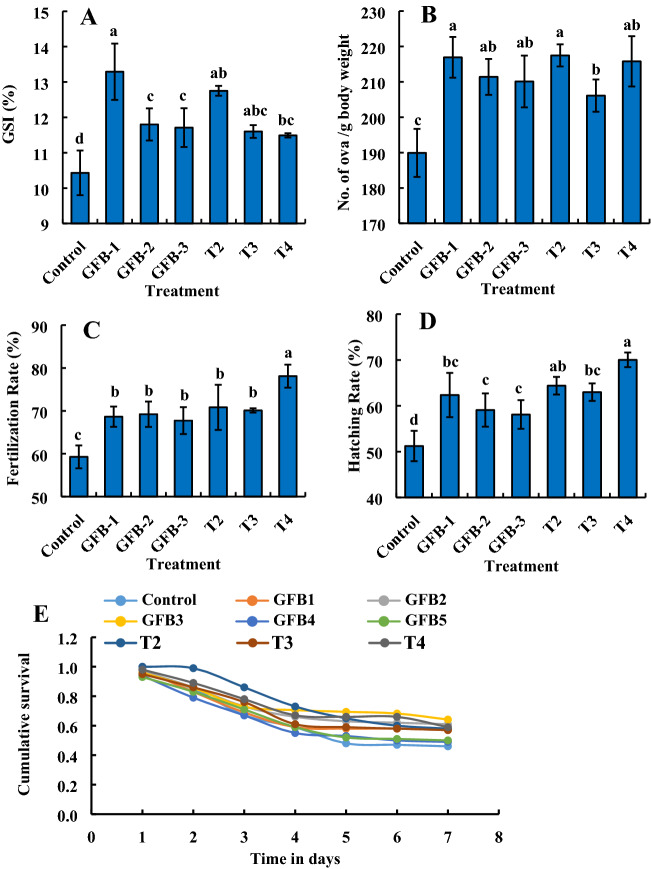
Comparative study between assessment of individual effects of five probiotic bacterial strains viz. GFB-1, GFB-2, GFB-3, GFB-4 and GFB-5 and consortium effects of five bacterial strains viz T2, T3 and T4 isolated from the gut of *B. gonionotus* on reproductive performances (A, B, C, D and E) of the host fish. One way ANOVA was performed for analyzing the data of three replicated experiment and data in column varies significantly in LSD at *P* < 0.05 (Statistix 10). Different letter bars indicate significant variations in GSI, no. of ova/ g body weight, fertilization rate, and hatching rate (n = 100) of hatchlings of the host fish in different groups by the isolates at *P* < 0.05 (Statistix 10). Cumulative survival of larva with a 95% CI was performed for survival analysis. Error bar =  ± SD; n = 24.

Significantly (*P* < 0.05) the highest GSI and number of ova/g body weight were found in fishes fed feed supplemented with GFB-1 compared to the fishes fed feed supplemented with T2, T3, T4, GFB-2, or GFB-3 (Fig. 12A, B). Interestingly, significantly (*P* < 0.05) the highest number fertilization rate, and hatching rate were found in fishes fed feed supplemented with a consortium of all five bacterial isolates at the highest dose of 3 × 1.35 × 10^9^ CFU kg^−1^ feed compared to the fishes fed feed supplemented with T2, T3, GFB-1, GFB-2 or GFB-3 (Fig. 12C, D). In addition, the Kaplan–Meier survival analysis indicated that GFB-3 treated larva exhibited the highest cumulative survival followed by GFB-2, T4, T2, GFB-1, T3, GFB-5, and GFB-4 (Fig. 12E).

### Probiotic bacteria improve the host–bacteria interactions

**Figure 13:**
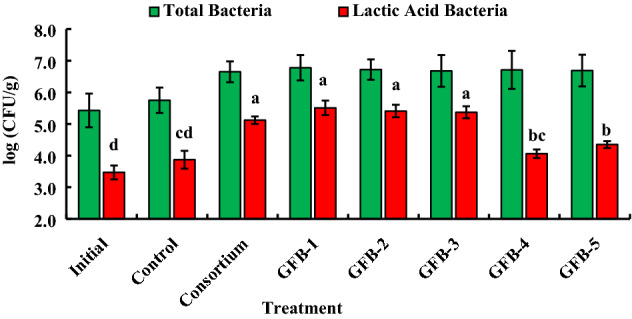
Assessment of consortium effects of five probiotic bacterial strains and individual effect of five probiotic bacterial strains viz*.* GFB-1, GFB-2, GFB-3, GFB-4 and GFB-5 isolated from the gut of *B. gonionotus* on the culturable autochthonous bacteria and autochthonous lactic acid bacteria levels (log CFU/g intestine) of the host. One way ANOVA was performed for analyzing the data of three replicated experiment and data in column varies significantly in LSD at *P* < 0.05 (Statistix 10). Different letter bars indicate significant variations in the culturable autochthonous bacteria and autochthonous lactic acid bacteria levels of the host fishes in different groups by the isolates at *P* < 0.05 (Statistix 10). Error bar =  ± SD; n = 9.

To investigate the microbiota status in the gut of *B. gonionotus*, we considered three fish from each replication. The highest number of lactic acid bacteria were log (5.51 ± 0.23) CFU/g, log (5.41 ± 0.2) CFU/g, log (5.37 ± 0.19) CFU/g, log (5.12 ± 0.12) CFU/g in GFB-1, GFB-2, GFB-3 and consortium (T2), respectively and the number of lactic acid bacteria was log (3.87 ± 0.28) CFU/g in the untreated control (Fig. 13). Interestingly, a consortium of all probiotics (T2), GFB-1, GFB-2, and GFB-3 significantly (*P* < 0.05) enhanced the growth of lactic acid bacteria in the gut of the host compared to GFB-4 and GFB-5 or untreated control (Fig. 13).

### Gut probiotic bacteria enhance the digestive enzyme activity

The effects of five gut bacterial isolates on the digestive enzyme activities were determined to evaluate their feasibility as probiotic candidates (Table 2). In this study, all five bacterial isolates enhanced protease and lipase activities. Among the bacterial isolates, significantly (*P* < 0.05) the highest protease activities were demonstrated by GFB-3 (8.60 ± 0.61 µg ml^−1^ h) followed by GFB-1 (8.43 ± 0.41 µg ml^−1^ h) and GFB-4 (7.60 ± 0.49 µg ml^−1^ h) (Table 2). Significantly (*P* < 0.05) the highest lipase activity was shown by the isolate GFB-1 (1.43 ± 0.03 µmol fatty acid ml^−1^) followed by GFB-2 (1.40 ± 0.03 µmol fatty acid ml^−1^) and significantly (*P* < 0.05) the lowest lipase activity was recorded for the isolate GFB-4 (1.27 ± 0.06 µmol fatty acid ml^−1^) (Table 2). All five bacterial isolates showed starch amylase hydrolysis activity (Table 2).

**Table 2:** The effects of the five gut probiotic bacteria on the digestive enzymes activity.

Isolates	Protease activity (µg ml^−1^ h)	Lipase activity (µmol fatty acid ml^−1^)	Starch amylase activity
GFB-1	8.43 ± 0.41^ab^	1.43 ± 0.03^a^	+
GFB-2	7.27 ± 0.29^ab^	1.40 ± 0.03^ab^	++
GFB-3	8.60 ± 0.61^a^	1.30 ± 0.03^bc^	++
GFB-4	7.60 ± 0.49^ab^	1.27 ± 0.06^c^	+++
GFB-5	7.20 ± 0.17^b^	1.30 ± 0.03^bc^	+

Values are expressed as mean. Different letters on the rows indicate significant difference by LSD test (*P* < 0.05); (n = 3); Inhibition zone > 3 =  +++, > 2 =  ++, > 1 =  +.

### Gut probiotic bacteria are susceptible to antibiotics

To find out whether the probiotic bacterial isolates had varying levels of susceptibility to commonly used antibiotics, the bacterial isolates were screened against 10 antibiotics using disk diffusion assay. Interestingly, all of the five probiotic bacterial isolates displayed susceptibility to all of the antibiotics except penicillin-G and amoxicillin (Table 3).

**Table 3:** Antibiotic susceptibility profile of five bacterial probiotic strains isolated from the gut of *B. gonionotus.*

Isolates	Inhibition zone ratio for tested antibiotics									
	Ampicillin (AMP)	Cefuroxime (CXM)	Nitrofurantoin (NIT)	Vanco-mycin (VA)	Penicillin-G (P)	Gentamicin (GEN)	Erythromycin (E)	Amoxicillin (AMX)	Cefradine (CH)	Levofloxacin (LE)
GFB-1	2.1 ± 0.1	4.5 ± 0.1	4.0 ± 0.0	3.7 ± 0.1	R	3.2 ± 0.0	R	R	5.2 ± 0.1	5.4 ± 0.1
GFB-2	R	2.3 ± 0.0	2.1 ± 0.1	4.0 ± 0.1	R	3.8 ± 0.1	4.3 ± 0.0	R	3.5 ± 0.0	5.8 ± 0.1
GFB-3	1.5 ± 0.0	R	4.0 ± 0.0	3.8 ± 0.0	R	4.2 ± 0.1	3.5 ± 0.1	1.4 ± 0.0	4.0 ± 0.0	6.7 ± 0.1
GFB-4	R	R	3.4 ± 0.1	2.9 ± 0.0	R	3.4 ± 0.0	1.2 ± 0.0	R	3.1 ± 0.1	3.8 ± 0.1
GFB-5	R	R	3.0 ± 0.0	3.0 ± 0.0	R	3.1 ± 0.1	2.2 ± 0.0	R	3.3 ± 0.0	4.0 ± 0.1

Ampicillin (25 μg disk^−1^), Cefuroxime (30 μg disk^−1^), Nitrofurantoin (30 μg disk^−1^), Vancomycin (30 μg disk^−1^), Penicillin-G (10 μg disk^−1^), Gentamicin (10 μg disk^−1^), Erythromycin (15 μg disk^−1^), Amoxycillin (30 μg disk^−1^), Cefradine (25 μg disk^−1^), Levofloxacin (5 μg disk^−1^), R = Resistant. Disk diameter is 6.0 mm. Data are presented as Mean ± SE. (n = 3).

### The cytotoxic and toxigenic effects of gut probiotic bacteria

The cytotoxic and toxigenic effect of the five bacterial isolates on the survival of *Artemia salina* nauplii were assessed to confirm their safety as probiotic bacteria. Interestingly, no mortality of *A. salina* was found in GFB-1, GFB-2, and GFB-3. However, GFB-4 and GFB-5 showed 20% ± 4.08 and 28% ± 8.5 mortality of *A. salina*, respectively.

## Discussion

In the present study, we isolated and identified five strains of probiotic bacteria viz*. E. xiangfangensis* (GFB-1), *P. stutzeri* (GFB-2), *B. subtilis* (GFB-3), *C. freundii* (GFB-4), and *P. aeruginosa* (GFB-5) from the gut of *B. gonionotus* collected from a fish farm of Bangladesh using classical microbiological methods and *16S rRNA* gene sequencing. Phenotypic characteristics, the enhancement of lactic acid bacteria and digestive enzyme activity, susceptible to antibiotic-resistant, histological parameters, and safety bioassay revealed that these five bacterial isolates are probiotic bacteria. Application of both consortium and individual bacteria with formulated fish feed significantly improved growth, hematological, and reproductive performances of the host fishes compared to the untreated control. Although enhancement of growth and disease resistance in fish treated with probiotic bacteria have been reported by several researchers^12,14–17^, this study for the first time demonstrated that gut probiotic bacteria such as GFB-1*,* GFB-2, GFB-3, GFB-4, and GFB-5 belonging to the genera of *Enterococcus, Pseudomonas, Bacillus*, and *Citrobacter* isolated from *B. gonionotus* significantly improved the growth, hematological and reproductive performances of the host fish.

An important finding of our study is that all five probiotic bacteria were isolated from the gut of the locally cultured fishes. The *E. xiangfangenesis* was first isolated from the traditional Chinese pickle as a human probiotic bacterium^28^. However, there is no report about its role on fish or other organisms. A further study is warranted to elucidate the underlying molecular mechanism of this fish growth-promoting effects by this probiotic strain of *E. xiangfangenesis*.

The probiotic effects of *Pseudomonas* bacteria on fishes are well known^29^. However, the *P. stutzeri* (GFB-2) isolated from the gut of *B. gonionotus* is a probiotic strain having positive effects on growth, hematological parameters, and reproductive performances of the host fish. The *P. stutzeri* was first isolated from the human spinal fluid, which was also found in diverse environments including the sediments of waterbodies^30^. Similarly, *P. aeruginosa* (GFB-5) is a Gram-negative bacterium widely distributed in soil and water^31^. However, in our study, both GFB-4 and GFB-5 did not give either beneficial or detrimental effects on the growth, hematological parameters and reproductive performances of the treated host fish.

The beneficial effects of fish probiotics belonging to the genus *Bacillus* on the induction of digestive enzymes, growth promotion, disease protection, and enhancement of host immunity have extensively been investigated^32^, and some of the probiotic strains of this genus have already been commercialized for the promotion of aquaculture industry^32^. It has been reported as a promising candidate of the probiotic bacterium for tilapia (*Oreochromis niloticus*)^33^. The species of the genus *Bacillus* are also known to produce diverse classes of antibiotics and antimicrobial enzymes that are reported to protect host organisms through antibiosis and induction of systemic resistance^34^. The *C. freundii* (GFB-4) is a Gram-negative and facultative anaerobic bacterium frequently found in water, soil, and in the intestine of animals^35^.

The highest weight gain was 89.58 ± 0.56 g in fish fed feed supplemented dose at 1.35 × 10^9^ CFU kg^−1^ feed for 60 days concurs with the results of Opiyo et al.^36^ who recorded high body weight of Nile tilapia fed on a diet supplemented *B. subtilis* at the dose of 10 g kg^−1^ feed. The higher growth reported in fish fed on a diet with the five probiotic strains in the present study is in agreement with Xia et al.^37^ who reported an increase in weight gain and lower FCR reported in *O. niloticus* fed on *B. cereus* NY5 and the mixture of *B. subtilis* and *B. cereus* NY5 at 1 × 10^9^ CFU/g feed for 4–8 weeks. These results might be implied that the oral administration of probiotics increased the activities of intestinal digestive enzymes^38^. In the current study, the probiotics tremendously enhanced the protease, lipase, and amylase activities in the gut of *B. gonionotus*. These digestive enzymes may be responsible for the hydrolysis of the major nutrients to hydrolysates that may be absorbed in the intestine. Our study also showed that fish fed supplemented with a consortium of probiotic strains at the dose of 3 × 1.35 × 10^9^ CFU kg^−1^ feed significantly reduced lower growth performances which are supported by Bagheri et al.^39^. The possible reason behind the increment of FCR with the increasing of probiotic doses due to the higher inclusion level of probiotic reduced the ileal digestibility and total tract apparent digestibility of nutrients compared to lower inclusion level of probiotics. The lower nutrient digestibility was due to the higher demand for nutrients by probiotic bacteria provided through the feed^40^. These results indicate that growth enhancement is postulated to exist via the colonization and activities of bacteria in the gut^38^. The isolated probiotic strains from the gut of *B. gonionotus* enhanced proteases, lipases, and starch amylases activity which conferred beneficial effects in the digestion process and aided in the growth performances of the fishes^38,41^. However, further studies are needed to test these hypotheses. A large body of literature supports these hypotheses. For example, a probiotic *Streptococcus* strain was supplemented to the diet of Nile tilapia which significantly enhanced the fish growth^42^. *Pediococcus acidilactici* evaluated as a probiotic strain improved intestinal health, growth performance, and feed utilization^43^. Similarly, Pirarat et al.^44^ reported that lactic acid bacteria from human origins accelerate the growth performance, gut mucosal, humoral, and cellular immune response of Nile tilapia. The homologous strains *Micrococcus luteus* and *Pseudomonas* spp. isolated from gonads and intestine of Nile tilapia positively increased growth performance and survival of the host fish^45^. *Lactobacillus acidophilus* and *Bacillus* spp., strains supplemented with diets showed significant positive effects on the growth parameters viz*.,* SGR, FCR, DGR, and survival and production of *Clarias gariepinus* and channel catfish^46^. Some studies showed that the mixture of optimum dosages of probiotic supplemented diets significantly enhanced the growth of fishes^36,37,39^, which are consistent with the findings of the present study. These indicate that the consortium of five probiotic bacteria at a lower dose of 1.35 × 10^9^ CFU kg^−1^ feed might be a potential dose for use in aquaculture.

In the present study, the bacterial probiotic strains both in consortium or individual (GFB-1, GFB-2, and GFB-3) are likely to modulate the intestinal microbiota by reducing the number of pathogenic bacteria which ultimately promoted healthy intestinal villi. Consequently, the gut probiotic bacteria can get opportunity to secrete by-products which can activate the digestive enzymes for digestion in the gut of fish. Similarly, Pirarat et al.^44^ reported that probiotic bacteria *L. rhamnosus* significantly improved the length of intestinal microvilli in *Oreochromis niloticus* which is consistent with the present study. These results suggest that the length of villi might likely able to increase the intestinal nutrient absorption area, which might contribute to an improvement in feed utilization and growth. However, the mechanism by which the tested probiotics improve intestinal absorption of fishes is not clear. Generally, the cells on the tip of these villi are continuously sloughed off and the renewal of the intestinal epithelium is reported to be extraordinarily high to replace the cells^47^. In our study, the probiotics showed a positive relationship with other lactic acid bacteria in the host gut. These lactic acid bacteria have beneficial effects on the gut microbiome and also inhibit the growth of various fish pathogens^15,17,19,37^.

The probiotic strains enhanced the number of RBC and the level of hemoglobin in the blood, consequently, the treated fishes achieved a sufficient amount of oxygen for their respiration. Moreover, these bacteria enhanced the amount of WBC which might be enhanced the immunity of fish resulting in the prevention of fish diseases^48^. The feeding of the probiotic bacterial strains isolated from the gut of *B. gonionotus* led to a significant increase in the number of erythrocytes as well as an increase in the white blood cells which helps in non-specific immunity via neutrophils and macrophages^49^. Application of the probiotic bacterial strains in formulated diets also enhanced glucose level, sugar level, and PCV level and these results suggested that fish might be consumed a lot of probiotic enriched feed^50,51^. Consequently, fish growth was enhanced without the attachment of harmful pathogens. Sampath et al.^52^ reported that the percentage volume of erythrocytes and total and different leucocyte counts in the blood represented a good health status of fish. In addition, the strains might induce the immune cells to enhance the non-specific immune responses^49,53^. Probiotic bacteria may also stimulate the proliferation of lymphocytes (both B and T cells) and immunoglobulin production in fishes^46,54^ that are consistent with our study. The consortium of probiotics significantly enhanced the number of WBC in *Cyprinus carpio*^55^, PCV value in *Clarias batrachus*^56^, but no change of hemoglobin level^55,56^. Further studies are needed to elucidate the mode of actions of beneficial effects of the gut probiotic bacteria shown in this study.

The results of this study demonstrate that incorporation of gut probiotics in feed favourably influenced the reproductive performance of *B. gonionotus* in terms of high GSI, high ova/g body, high fertilization rate, high hatching rate and high survival of fry. Although the mechanism of beneficial effects of the tested probiotic bacteria is not clear from the current study, the probiotic bacterial strains might be involved in the inhibition of secretion of dopamine hormone which is an inhibitor of gonadotropin-releasing hormone (GnRH) during the breeding season^57^. Probiotic bacteria established in the gut enhance broodstock and larval nutrition by synthesizing essential nutrients (proteins and essential fatty acids) and enzymes (amylase, protease and lipase)^49^. Proteins and fatty acids are very important constituents of the yolk and their presence in diet consequently support good oocytes development and maturation and a higher rate of vitellogenesis^58^. Besides the regulation of reproductive physiology, essential fatty acids also supply energy to sustain the spawning activity. Probiotic bacteria also produce B group vitamins^59^, and the production and supply of B vitamins and certain unknown stimulants^60^ could have played a key role in the elevated reproductive performances of the probiotic feed fed fish. Ghosh et al.^18^ isolated *B. subtilis* from the intestine of *Cirhinus mrigala* and applied it with the diet of four species of ornamental fishes in a 1-year feeding experiment. They found that *B. subtilis* increased GSI, fecundity, survival, and production of fry from the female of all tested fishes which is consistent with the findings of our study. Abasali and Mohamad^61^ also recorded an increase in the GSI and the production of fingerlings of females in reproductive age and the fecundity in *X. helleri* spp. supplemented with commercial probiotic-containing four species of lactic acid producing bacteria. Improvement of reproductive performances of fishes by the application of various probiotic bacteria has been reported by many researchers^62,63^.

Fish fed supplementation of the consortium of five probiotics bacteria at high dosage showed significantly the highest fertilization rate, hatching rate, and survival rate compared to GFB-1, GFB-2, and GFB-3 that coincided with the results of Chitra and Krishnaveni^64^. In our study, GFB-1, GFB-2, and GFB-3 may reduce fat accumulation in the liver which is accumulated from the formulated feed ingredients soybean meal and mustard oil cake. These results indicate that the gut probiotic strains treated with formulated feeds would be safe for fish health as well as beneficial for human health. Similarly, Ruiz et al.^23^ reported that probiotic bacterium *Lactobacillus plantarum* improved hepatic function and promote liver restoration in Nile tilapia. Moreover, histological analysis of the liver of sea bass fed different levels of yeast probiotic extract showed steatosis with fat degeneration, whereas liver morphology was considerably improved with yeast probiotic extract supplementation^65^.

In our study, most of these isolated bacterial strains are susceptible to antibiotics, suggesting that these probiotic bacteria could be used as an alternative to antibiotics in aquaculture^11^. In this regard, the probiotic bacteria, GFB-1, GFB-2, and GFB-3 might be potential aquaculture candidate to control fish diseases^16^. In the current study, the probiotic bacterial strains GFB-1, GFB-2, and GFB-3 did not show any significant cytotoxic and toxigenic effects on the survival of *A. salina* nauplii. There are no legal limitations for research on probiotics in Bangladesh. However, legal approval will be needed before the application of these bacteria for commercial use.

In conclusion, the present study demonstrated that dietary supplementation of gut probiotic bacteria isolated from *B. gonionotus* promoted growth, hematological parameters, and reproductive performances of the host fishes. The gut bacteria, *E. xiangfangensis* (GFB-1), *P. stutzeri* (GFB-2), and *B. subtilis* (GFB-3) should be considered as promising individual candidates for promoting sustainable aquaculture in Bangladesh. These findings also suggested that a consortium of probiotic bacterial strains such as GFB-1, GFB-2, and GFB-3 supplemented diets could be developed for promoting the growth, hematological parameters, and reproductive performances of silver barb fishes in aquaculture. This is the first description of molecular identification and demonstration of beneficial effects of some gut probiotic bacteria that promoted growth, histological parameters, hematological parameters, and reproductive performances of the *B. gonionotus* fishes. Further studies should be focused on the elucidation of underlying mechanisms of the beneficial effects and safety issues of these gut probiotic bacteria on the host fishes. A large-scale field trial of these gut probiotic bacteria is needed before recommending them for practical application in aquaculture.

## Methods

### Collection of experimental fish

A total of 480 (average 65.6 ± 0.8 g) experimental fish (*B. gonionotus*) were collected from an aquaculture farm of Trishal, Mymensingh, Bangladesh, and transported to the wet laboratory of Dept. of Genetics and Fish Breeding, Faculty of Fisheries, Bangabandhu Sheikh Mujibur Rahman Agricultural University, Gazipur, Bangladesh with the provision of continuous aeration. Handling, holding and releasing of all experimental fish were done following Canadian Council on Animal Care guidelines^66^. Briefly, the fish were stocked in the circular plastic tanks (500 l) with aerators and acclimatized for 15 days in the wet laboratory according to Mohapatra et al.^51^. Water change was done every 3 days interval and uneaten feeds were collected. Water quality parameters such as, pH, dissolve oxygen, and temperature were routinely measured to maintain the health of fish. During the conditioning, the fish were fed the commercial diet (Mega Feed Company Ltd, Bangladesh; containing 30% protein and 8% fat) at 5% of body weight until the feeding trial started.

### Euthanasia methods

During experiment, fish were anesthetized and euthanized by using clove oil (Sigma C8392). Clove oil solution was prepared according to Fernandes et al.^67^. Briefly, pure clove oil was first dissolved in ethyl alcohol in 1:9 ratio (clove oil: ethyl alcohol). This solution then diluted in water in order to obtain concentrations of 0.05 ml (50 mg), and 0.20 ml (200 mg) of clove oil per 500 ml of water. For hematological study, experimental fish were anesthetized by using 0.05 ml clove oil per 500 ml of water. For histological, reproductive and intestinal microflora study, fish were euthanized by using 0.20 ml of clove oil per 500 ml of water, and death was confirmed by the destruction of the brain^66,67^.

### Collection of probiotic samples from the gut of *B. gonionotus*

Healthy *B. gonionotus* fish were collected from an aquafarm from Trishal, Mymensingh, Bangladesh for probiotic isolation. Probiotic bacteria were isolated from the gut of healthy *B. gonionotus* according to Wanka et al.^19^ with some modifications. Briefly, ten fish were sacrificed upon anesthetization with clove oil (0.20 ml per 500 ml of water) ^67^ according to the guidelines on humane killing of fish^66^. The abdomens of fish were cut aseptically by sterile scissors and the gut was taken out with care to avoid any distortion of gut and contamination with blood. The gut was cut into small pieces and rinsed with 0.9% (w/v) saline solution and placed in a conical flask containing 10 ml distilled water. The sample was stirred with a stirrer to make a homogenous solution.

### Isolation of probiotic bacteria from the gut of *B. gonionotus*

One gram of sample was diluted in 10 ml sterilized water and inoculated on De Man, Rogosa, and Sharpe agar (MRS) media (Himedia, India) for *Lactobacillus* spp using spread culture method at Laminar Air Flow cabinet. The agar plates were incubated at 28 °C for 24 h in an incubator and the colony characteristics were observed carefully to choose desired colonies. Pure culture of each isolate was done by the streak culture method. The isolates were routinely sub-cultured on NA (Nutrient agar) plates and incubated at 28 °C and stored in a freezer at -20 °C supplemented with 10% glycerol. Selective colonies were characterized and identified for their colony and biochemical and physiological characteristics^68^.

### Molecular identification of probiotic strains

Genomic DNA of the selected isolates was extracted by using a commercial GenJET genomic DNA purification kit (Thermo Fisher Scientific, USA) following the manufacturer’s protocols. The extracted DNA was used for PCR amplification and stored at − 20 °C for further use. DNA was amplified by using universal primer 8F (5′-AGAGTTTGATCCTGGCTCAG-3′) and 1492R (5′-GGATACCTTGTTACGACTT-3′). The PCR amplification was done according to Rahman et al.^69^. The PCR amplification condition was done by an initial denaturation at 94 °C for 5 min; 35 cycles of a denaturation at 94 °C for 1 min, an annealing at 57 °C for 40 s and an extension at 72 °C for 1 min and a final extension step at 72 °C for 10 min. Then the PCR amplicons were purified by using a commercial kit (Thermo Fisher Scientific, USA) and *16S rRNA* gene sequencing was done from the National Institute of Biotechnology, Dhaka, Bangladesh. The sequence data were extracted by using MEGA7 software as FASTA format^70^. Homology of the *16S rRNA* gene sequences of the strains were studied by using the Basic Local Alignment Search Tool (BLAST) program of the National Centre for Biotechnology Information (NCBI) and phylogenetic analysis was done by using the MEGA7 software following the neighbor-joining method^70^.

### Preparation of probiotic strains

Probiotic strains were prepared according to Xia et al.^37^. Briefly, the five probiotic strains isolated from *B. gonionotus* gut were identified based on their morphological, physiological, and biochemical characteristics, as well as *16S rRNA* gene sequencing. For the preparation of probiotic strains, each strain was cultured in 1 l nutrient broth in an orbital shaker and incubated at 28 °C for 24 h. Then the broth media was centrifuged at 8000×*g* for 5 min. The pelleted probiotic bacterial strains were collected and washed twice in sterile water. The pellets were then suspended in sterile distilled water and were added to the dough. A spread plate technique was used to assess the viability of cells according to the cell concentrations measured at OD600. All cell suspension OD600 values were adjusted to an adequate value (CFU ml^−1^) for further experiments. The broth cultures with the five probiotic strains were carried out aseptically in the advanced molecular laboratory at a controlled temperature of 28 °C^27,71^.

### Acidic pH tolerance test and preparation of simulated gastrointestinal juice of host

Acidic pH tolerance test was performed as previously described by Guerra et al.^71^ with a few modifications. The five gut probiotic bacteria were grown in MRS broth in an orbital shaker and incubated at 28 °C for 24 h and the cultures were centrifuged at 8000×*g* for 5 min at 4 °C. The pellets were washed and suspended in sterile phosphate-buffered saline solution (PBS). Each probiotic strain was diluted 10^–2^ in sterile PBS at pH 1.0, 2.0, 3.0, 4.0 and 5.0. After incubating 1, 2, and 4 h and serial decimal dilutions in sterile PBS were prepared for determining the total viable cell number. The experiment was repeated twice and each reading shows the mean of three observations. Gastrointestinal juices were prepared fresh by dissolving pepsin (Thermo Fisher, USA) from *B. gonionotus* stomach mucosa (3 g l^−1^) in sterilized saline solution (5 g l^−1^) followed by Charteris et al.^25^. Subsequently, the pH of the gastrointestinal preparation was adjusted to 2.0 with 12 M HCl.

### Exposure of gut probiotics to simulated gastrointestinal juice and total viable counts

The five gut probiotic bacteria were exposed to simulated gastrointestinal juice at 28 °C for 24 h according to Guerra et al.^71^. Briefly, a 1-ml aliquot of each culture was centrifuged at 5000×*g* for 10 min at 4 °C and washed three times in sterile PBS. The washed cells were resuspended in 300 µl PBS. The total viable counts of the washed cells suspension were determined above prior to assay of gastrointestinal transit tolerance. The tolerance of five probiotic bacteria to simulated gastrointestinal juices was determined by mixing 0.2 ml of each washed cell suspension with 1 ml of gastric juice. After brief vortexing, the mixtures were incubated at 28 °C. When assaying gastric transit tolerance, aliquots of 0.1 ml were removed after 5, 40, and 180 min for determination of total viable count. The experiment was repeated twice and each reading represents the mean of three observations.

### Experimental feed preparation

The probiotic supplemented dough containing indigenous dry ingredients was prepared according to Abdel-Tawwab et al.^72^. All fresh diet ingredients were purchased locally and diets were formulated as shown in Table 4. The ingredients were mixed properly aseptically with adding sterile water. A large amount of dough was prepared and divided into different portions for inoculation with the same volume of sterile water. The control diet was not supplemented with any bacteria. For probiotic supplemented feed preparation, 50 ml of probiotic bacterial suspension were centrifuged at 6000×*g* for 10 min. After discarding the supernatant, bacterial pellets were mixed properly with sterile physiological saline (0.85% NaCl) and added in 1 kg of dough, and aseptically mixed properly in a controlled environment. Then aseptically feeds were prepared as pellet form using sterilized aluminum wire sieve, dried at room temperature in a controlled laboratory condition, packed in sterilized zip-lock bags, and stored at 4 °C up to 7 days. Probiotic formulated feed was prepared weekly by maintaining these processes and the viable count of bacteria in feed was assessed followed by Guerra et al.^71^. Briefly, the survival of the probiotic bacteria in formulated fish feed was assessed using the broth cultures of each probiotic bacteria through the pour plate method. The formulated feed was mixed with the corresponding broth culture (20 ml kg^−1^ of feed) and stored at room temperature. Daily, duplicate samples (10 g) of probiotic supplemented fish feed were mixed at 1:10 with sterile PBS and vortexed for 2 min. Both samples were serially diluted using sterile PBS and each dilution was plated in triplicate in MRS agar. Plates were incubated at 25 °C for 48 h. Incubated plates were observed for the optimum number of CFU, between 30 and 300 colonies per plate. The results were expressed as the number of colonies counted per gram (wet weight) of feed. Dough contained 37% protein for treating all experimental fish for both consortium applications of probiotic bacteria and individual application of probiotic bacteria according to proximate composition analysis of dough by AOAC^73^ as shown in Table 5.

**Table 4:** Composition of experimental feed supplementing with the graded level of consortium and individual of probiotic bacterial strain for the rearing of *B. gonionotus.*

Inclusion level (%)	Ingredients							
	Fish meal^1^	Soybean meal^2^	Mustard oil cake^3^	Rice bran	Wheat bran	Wheat flour	Vitamin mineral premix^4^	Probiotic bacteria (CFU/Kg feed)^5^
**Consortium**								
Feed 1 (T1)*	40.00	19.58	15.00	10.00	10.00	4.00	1.00	0
Feed 2 (T2)	40.00	19.58	15.00	10.00	10.00	4.00	1.00	1.35 × 10^9^
Feed 3 (T3)	40.00	19.58	15.00	10.00	10.00	4.00	1.00	2 × 1.35 × 10^9^
Feed 4 (T4)	40.00	19.58	15.00	10.00	10.00	4.00	1.00	3 × 1.35 × 10^9^
**Individual probiotic**								
GFB-1	40.00	19.58	15.00	10.00	10.00	4.00	1.00	1.62 × 10^9^
GFB-2	40.00	19.58	15.00	10.00	10.00	4.00	1.00	1.43 × 10^9^
GFB-3	40.00	19.58	15.00	10.00	10.00	4.00	1.00	1.06 × 10^9^
GFB-4	40.00	19.58	15.00	10.00	10.00	4.00	1.00	1.5 × 10^9^
GFB-5	40.00	19.58	15.00	10.00	10.00	4.00	1.00	1.13 × 10^9^

*Denotes control

^1^Locally purchased, crude protein 70%, crude lipid 9%

^2^Mega Feed Limited, Bangladesh, crude protein 49%, crude lipid 20%

^3^Locally purchased, crude protein 40%, crude lipid 20%

^4^Vitamin premix (mg/kg diet): thiamin, 25; riboflavin 20; pyridoxine 21; cyanocobalamine, 0.03; folic acid 5; calcium pentothenate, 45; inositol, 100; niacin 100; biotin 0.1; starch, 3400; ascorbic acid, 100; Vitamin A, 100; Vitamin D, 20; Vitamin E, 50; Vitamin K, 12

^5^Extracted and isolated from gut of *B. gonionotus*.

**Table 5:** Analysis of proximate composition of control feeds, consortium and individual of the graded level of probiotic treated feeds (dry matter basis) for the rearing of *B. gonionotus.*

Treatment	% Dry matter	% Lipid	% Protein	% Ash	% Crude fiber	% Carbohydrate
**Consortium probiotic bacteria**						
*T1	88.92	10.07	37.27	14.21	6.13	32.32
T2	88.75	9.99	37.11	14.37	6.09	32.44
T3	88.74	10.11	37.13	14.51	5.99	32.26
T4	88.23	10.17	37.04	14.23	6.20	32.36
**Individual probiotic bacteria**						
Control	88.93	10.10	37.20	14.22	6.14	32.34
GFB-1	88.78	10.02	37.13	14.30	6.10	32.45
GFB-2	88.81	10.12	37.14	14.41	6.03	32.30
GFB-3	88.23	10.18	37.05	14.24	6.21	32.32
GFB-4	88.92	10.13	37.06	14.37	6.04	32.31
GFB-5	88.85	10.03	37.14	14.31	6.12	32.40

*Denotes control.

The control diet was formulated using indigenous ingredients. The dough prepared by adding the required amount of water to these ingredients was steam sterilized and incorporated with a commercial vitamin-mineral mix at 1% (v/w). Colony-forming units (CFU) were calculated according to Mohapatra et al.^51^. The isolated probiotic strains viz. GFB-1, GFB-2, GFB-3, GFB-4 and GFB-5 were mixed at ratio of 1:1:1:1:1 with the formulated diets. The total well-mixed feed ingredients without probiotics were divided into four portions and mixed with the addition of probiotics concentration of 0 (Control), 1.35 × 10^9^, 2 × 1.35 × 10^9^, and 3 × 1.35 × 10^9^ CFU kg^−1^ feed to determine the consortium effect of isolated probiotic strains. Again, the feed ingredients were divided into six portions and mixed with the addition of probiotics concentration of 0 (Control), 1.62 × 10^9^ with GFB-1, 1.43 × 10^9^ with GFB-2, 1.06 × 10^9^ with GFB-3, 1.5 × 10^9^ with GFB-4, and 1.13 × 10^9^ CFU kg^−1^ feed with GFB-5 to determine the individual effect of isolated probiotic strains at different concentration^50^.

### Experimental design and feeding trial

To assess the consortium effect, twelve plastic tanks (500L) were divided into four treatments such as T1 (control) (0), T2 (1.35 × 10^9^), T3 (2 × 1.35 × 10^9^), and T4 (3 × 1.35 × 10^9^) CFU kg^−1^ feed with three replicates. A total of 192 uniformly sized mature fish were randomly distributed in four treatments and the stocking density was maintained at 16 fish/tank (male: females at 1:3) following a completely randomized design. The fishes were acclimatized with commercial feeds for 15 days. After the acclimatization period, treatment T1 was fed with the control diet without probiotics, and treatments T2, T3, and T4 were fed with a consortium of five isolated probiotic strains for an experimental period of 60 days.

To determine the individual effect of probiotic strains, 18 tanks (500 l) were divided into six groups (each had three replicates) including one control group and five individual probiotic strains treatment groups. Similar size 288 mature fish were randomly distributed in six treatments with a stocking density of 16 fish/tank. Treatment was fed with 0 CFU (control), GFB-1 with 1.62 × 10^9^, GFB-2 with 1.43 × 10^9^, GFB-3 with 1.06 × 10^9^, GFB-4 with 1.5 × 10^9^, and GFB-5 with 1.13 × 10^9^ CFU kg^−1^ feed for an experimental period of 60 days. Fish hand-fed daily at 2.5% of the total biomass, twice daily at 0900 h and 1900 h for a period of 60 days. Feed adjustments were done for each tank every 15 days after sampling. The dough was prepared every 7 days interval. The uneaten feeds were collected during water exchange.

### Growth parameters

In order to assess the growth parameters, the fish samples were collected from a commercial aqua farm of Trishal, Mymensingh, Bangladesh. A total of 48 fish (each replication contain 16 fish) were taken for each treatment. The growth of *B. gonionotus* was evaluated in terms of weight gain, specific growth rate (%) (SGR (%/day) and feed conversion ratio (FCR). Sampling was performed every 15 days interval.

Weight Gain: The weight gain was calculated by using the formula:

$$ {\text{Weight}}\,{\text{gain}}\left( {\text{g}} \right)\, = \,{\text{Mean}}\,{\text{final}}\,{\text{weight}} - {\text{mean}}\,{\text{initial}}\,{\text{weight}}{.} $$

Specific growth rate:

$$ {\text{ SGR }}\left( {{\text{\% day}}} \right) = \frac{{{\text{ lnW}}_2{ } - {\text{lnW}}_1}}{{{\text{T}}_2{ }{-}{\text{T}}_1}} \times 100 $$

where lnW_1_ = The initial live body weight (g) at time T_1_ (day); lnW_2_ = The final live body weight (g) at time T_2_ (day).

Feed conversion ratio: The FCR was calculated by using the formula:

$$ {\text{FCR}} = {\text{Feed}}\,{\text{fed/Gain}}\,{\text{in}}\,{\text{weight}}\,{\text{of}}\,{\text{fish}} $$

### Histological analyses of intestine and liver of the probiotic treated silver barb

At the end of the growth trial experimental period (60 days), fish were collected for the histological study. The experimental fish were humanely killed by using clove oil (0.20 ml per 500 ml of water)^67^, and death was confirmed by the destruction of the brain^66^. Six fishes from each treatment were anesthetized with the clove oil and gradually sacrificed to collect intestine and liver for histological study^67^. By sacrificing fish, the whole liver and part of the intestine from each fish were dissected carefully, cut to separate each other, and stored in bouins solution for 24 h. Then, these samples were dehydrated in ascending grades of alcohol and cleared in xylene. The fixed tissues were embedded in histoparaffin (Paraplast plus; Sigma-Aldrich) and sections (7 µm) were cut using a microtome (CUT-5602, Germany). Then the sections of intestinal villi and liver were selected and stained with Delafield’s hematoxylin–eosin for observation under a light microscope (DM 100; Leica, Wetzlar, Germany). Ten slides were prepared from the intestine of each fish through histological method. Each slide contained ten intestinal tissue sections. Then the slides were observed under a trinocular microscope. Images were captured using a digital camera (DFC 290, Leica) and the villi length of the intestine was measured using AmScope software (Version 3.7; Carl Zeiss Primo Star, Germany).

### Measurement of hematological parameters

Blood samples were collected from the experimental fish according to Canadian Council on Animal Care^66^. At the end of the growth trial experimental period (60 days), a total of 27 fish from each treatment were anesthetized with the clove oil (0.05 ml per 500 ml of water) for hematological analysis^67^. Blood was collected from fish using a 3 cc syringe containing 10% blood anti-coagulant (EDTA) inserted into the caudal peduncle region to drag out blood. The blood was transferred to a test tube coated with EDTA, and stored at − 30 °C until use. Red blood cells (RBCs) and white blood cells (WBCs) were counted using an improved Neubauer hemocytometer (MarienFeld Company Germany) under the light microscope (DM 100; Leica, Wetzlar, Germany) according to Shah and Altindağ^74^. In order to measure hemoglobin, fresh blood was collected from fish from each treatment and was poured in the edge of a strip of hemoglobin meter before the coagulation of blood. The glucose level of blood was measured through a glucose meter from the sample. To measure packed cell volume (PCV) (%), blood was taken in a capillary tube at the marked level and sealed with gum. The capillary tube was placed in the rotor of hematocrit machine at a sealed point outward direction and the machine was allowed to run for 5 min at 15,000 rpm. The length of the blood cell was measured by hematocrit measuring scale and multiplied the recorded value with the concentration of blood. Packed cell volume (PCV) (%) = Hematocrit value × blood concentration × 100.

### Measurement of reproductive parameters of the host fish

At the end of the experiment period (60 days) of the assessment of the effects of probiotic bacteria on growth performances, the same fish was used for the measurement of the reproductive parameters. A total of 96 mature fish (72 females and 24 males) were selected for the assessment of consortium effect of probiotic and a total of 144 mature fish (108 females and 36 males) mature fish were selected for the assessment of the individual effect of probiotic on reproductive performances. A total of 24 mature fish were selected from each treatment and matured six females and two male broodstocks were selected from each replication. Sex was identified based on the external morphological characteristics^75^. Then eight fish (male:female = 3:1) from each replication were transferred into the individual holding tank and acclimatized for 2 days. The selected fishes were weighed by an electronic balance in g. Then three females and one male were stocked in each separate 50 l plastic bowls for 2 h. In order to observe the induced breeding of *B. gonionotus*, ovaprim (at the rate of 0.5 and 0.25 ml kg^−1^ body weight for females and males respectively) was used as an inducing agent. The extract of ovaprim was injected near the pectoral fin. The females were checked for ovulation after 5 h of injection in the bowl. The ovulated eggs of females were collected by stripping. Before stripping, the individual weight of females was recorded. The desired latency period of this fish was 8 h for control and 6 h for probiotic treated treatments. Then the females were stripped individually into the dry and preweighed beaker to record the stripped ova weight. No. ova/g body weight and percent ovulation was calculated according to Sahoo et al.^76^. The milt was collected from the male by stripping and mixed with eggs by gentle stirring with a feather. Then 5 ml water was added to the egg-sperm mixture to activate the sperms to fertilize the eggs. The gonadosomatic index (GSI %) was calculated by the following formula according to Sahoo et al.^76^.

$$ {\text{GSI}}(\% ) = \frac{{{\text{ Gonad }}\,{\text{weight}}}}{{{\text{Body}}\,{\text{weight}}}} \times 100 $$

A portion of fertilized eggs from an individual female of each treatment was homogeneously spread on three separate plastic bowls (32 cm diameter) and incubated. The fertilization rate was calculated according to Adebayo and Popoola^77^.

$$ {\text{Percent}}\,{\text{fertilization}}: = \frac{{{\text{No}}.\,{\text{of}}\,{\text{fertilized}}\,{\text{eggs}} \times 100}}{{{\text{ Total }}\,{\text{no}}.\,{\text{of }}\,{\text{eggs }}\,\left( {{\text{fertilized}} + {\text{unfertilized}}} \right)}} $$

The hatching rate was calculated according to Haniffa and Sridhar^78^

$$ {\text{Percent}}\,{\text{hatching}}: = \frac{{{\text{No}}.\,{\text{of}}\,{\text{ eggs}}\,{\text{ hatched}}}}{{{\text{Total }}\,{\text{no}}.\,{\text{of}}\,{\text{ eggs}}}} \times 100 $$

### Measurement of survival rate of larvae of the host fish

From the third day of hatching, the larvae were provided hard-boiled egg yolk. They were reared for seven days to observe the effect of the identified probiotic strains on their survivability as the larvae produced from the broods were maintained under different dietary levels of probiotic strains. Twelve plastic bowls for the consortium of probiotic strains and eighteen plastic bowls for individual probiotic strain each of 10 l capacity were divided into four groups and six groups corresponding to four treatments and six treatments respectively and each of the bowl was stocked with 100 larvae as a stocking rate of 10 larvae/l. The continuous water flow of nearly equal rate was maintained in all the bowls. The larvae were provided with live feed after the third day of hatching and administered thrice a day*.*

$$ {\text{Percent}}\,{\text{survival}}\,{\text{of}}\,{\text{larvae}} = \frac{{{\text{No}}.{\text{of larvae alive}}}}{{{\text{Total no}}.{\text{of larvae stocked}}}} \times 100 $$

### Assessment of gut microbiota of the host

Gut microbial flora was analysed according to Hoseinifar et al.^79^ with some modifications. In order to determine the total viable heterotrophic aerobic bacteria and lactic acid bacteria of control fish collected from the same aquafarm of Trishal, Mymensingh, Bangladesh. The study was accomplished at the start of the feeding trial by random sampling of 24 fish. The experimental fish were humanely killed by anesthetic overdose using clove oil (0.20 ml per 500 ml of water)^67^. In addition, at the end of the feeding trial of growth performances, 9 fish from each treatment (3 fish from each replication) by random sampling were examined to assess the effect of probiotic bacteria on gut microbiota.

### Evaluation of the gut probiotic bacteria on the activity of the digestive enzymes

Protease activity of the selected isolates was determined according to the method stated by Söderhäl and Unestam^80^. The lipase activity of probiotic bacteria was determined as described by Cordenons et al.^81^. In the starch amylase test, the test bacteria were grown on nutrient broth agar plates containing starch (5%). If the bacteria have the ability to hydrolyze starch transparent clear zones were formed around the colonies while the rest of the plate showed no clear zone.

### Assessment of antimicrobial resistances of probiotic bacterial strains

The antibiogram profile of the five bacterial isolates against 10 (Liofilchem, Italy and Himedia, India) was determined by Kirby–Bauer disc diffusion assay^82^. In a brief, isolated bacteria were inoculated into the nutrient broth and incubated at 28 °C for 24 h and the visual density of the broth was compared with 0.5 Mcfarland standard. Around 30 μl of broth of individual isolates were spread on iso sensitest agar media (Micro Master, India) by a sterile “L” shaped glass rod. Then the 10 antibiotic disks were placed on the agar plate by a sterile forceps and pressed gently with the forceps to ensure complete contact with the agar surface. After 24 h incubation at 28 °C, the zone around the disk was measured by a measuring scale. A clear zone of inhibition around the antibiotic disk indicated that the bacteria were susceptible to the antibiotic and no zone indicated that the bacteria were resistant to the antibiotic.

### Assessment of the cytotoxic and toxigenic potential of gut probiotic bacteria

The cytotoxic and toxigenic effect of five bacterial probiotic strains was determined through brine shrimp (*Artemia salina*) nauplii according to Lieberman^83^. The eggs of artemia were kept in brine with a constant oxygen supply for 48 h for hatching. Then the nauplii were used for the experiment. The bacterial probiotic cell extract was prepared through broth media. Then 1 ml bacterial cell-free extract added 1 ml of nauplii solution containing petri plate (100 cell/1 ml). The survivor nauplii were counted under a stereo microscope after 24 h.

### Water quality parameters

Water temperature, dissolved oxygen (DO), and pH of water in each replication under each treatment were recorded every day. Temperature and DO were measured by a digital Thermometer and DO meter (LUTRON PDO-519, TAIWAN). The pH was measured by a portable digital pH meter (EZODO, pH 5011). The water quality parameters in the range of pH 7.7–8.1, DO 5.6–5.8 mg l^−1^ and temperature 27–30 °C throughout the experimental period were maintained^84,85^.

### Statistical analysis

Data of weight gain, specific growth rate, intestinal villi length, gut microbiota, enzyme activity, gonadsomatic index, ovulation rate, fertilization rate, and hatching success were collected during the study period and statistically analysed using one-way analysis of variance (ANOVA) to test the significant results (*P* < 0.05) between means and the mean values were separated by LSD (least significance difference) posthoc statistic. Standard deviation (± SD) was calculated to identify the range of means. All statistical analyses were performed with the aid of the computer software Statistix 10.0 version. Power analysis was performed to check the statistical validity of sample size. The typical power analysis for an ANOVA was performed using G*Power version 3.0.10 according to Faul et al.^86^. Cumulative survival of larva were analysed through Kaplan–Meier survival analysis in Microsoft Office Excel version 2016 according to Jager et al.^87^. Weight gain data collected were repeated statistically analysed using ANOVA to test significance results (*P* < 0.05) between means. The standard error (± SE) was calculated to identify the range of means. These statistical analyses were performed with the aid of the computer software SPSS 26.0 version.

## Ethical approval

The use of animals was kept to an absolute minimum required to achieve statistical significance for validation purposes. All procedures were conducted in accordance with the United Kingdom Animal (Scientific Procedures) Act 1986, approved by Ethical Review Committee (ERC) of the Institute of Biotechnology and Genetic Engineering (IBGE), Bangabandhu Sheikh Mujibur Rahman Agricultural University (BSMRAU), Gazipur-1706, Bangladesh and conducted under the authority of the project Licence BSMRAU/IBGE/002. The study was done in compliance with the ARRIVE guidelines 2.0^88^. Statements are available as supplementary materials.

## Supplementary Information

Supplementary Information 1.

Supplementary Information 2.
