# Supplementary material for: Gut probiotic bacteria of Barbonymus gonionotus improve growth, hematological parameters and reproductive performances of the host
Source: Sci Rep. 2021 May 21;11:10692. doi: 10.1038/s41598-021-90158-x (PMC8140159; doi:10.1038/s41598-021-90158-x)
Supplement: Supplementary file 1 — Supplementary Information 1. [file 41598_2021_90158_MOESM1_ESM.docx]

**Supplementary materials for**

**Gut Probiotic Bacteria of *Barbonymus gonionotus* Improve Growth, Hematological Parameters and Reproductive Performances of the Host**

Mohammad Abdus Salam^1^*, Md. Ariful Islam^1^, Sulav Indra Paul^2^, Md. Mahbubur Rahman^2^, Mohammad Lutfar Rahman^1^, Fatama Islam^1^, Ashikur Rahman^2^, Dinesh Chandra Shaha^3^, Md Shah Alam^4^ & Tofazzal Islam^2^

^1^Department of Genetics & Fish Breeding, Faculty of Fisheries, Bangabandhu Sheikh Mujibur Rahman Agricultural University, Gazipur-1706, Bangladesh.

^2^Institute of Biotechnology and Genetic Engineering, Bangabandhu Sheikh Mujibur Rahman Agricultural University, Gazipur-1706, Bangladesh.

^3^Department of Fisheries Management, Faculty of Fisheries, Bangabandhu Sheikh Mujibur Rahman Agricultural University, Gazipur-1706, Bangladesh.

^4^Aquaculture Program Center for Marine Science, University of North Carolina Wilmington

601, S. College Rd. Wilmington, NC-28403, USA.

*Corresponding author: Tel: +88-0175-711-1593; Fax: +88-(02) 920-5310-14-2268

E-mail: [salamfish@bsmrau.edu.bd](mailto:salamfish@bsmrau.edu.bd)

**ARRIVE Essential 10**

**Item 1. Study design**

**For each experiment, provide brief details of study design including:**

**1a. The groups being compared, including control groups. If no control group has been used, the rationale should be stated.**

**Statement:** To assess the consortium effect, twelve plastic tanks (500L) were divided into four treatments such as T1 (control) (0), T2 (1.35 ×10^9^), T3 (2×1.35 ×10^9^), and T4 (3×1.35 ×10^9^) CFU kg^-1^ feed with three replicates. A total of 192 uniformly sized mature fish were randomly distributed in four treatments and the stocking density was maintained at 16 fish/tank (male: females at 1:3) following a completely randomized design. The fishes were acclimatized with commercial feeds for 15 days. After the acclimatization period, treatment T1 was fed with the control diet without probiotics, and treatments T2, T3, and T4 were fed with a consortium of five isolated probiotic strains for an experimental period of 60 days.

To determine the individual effect of probiotic strains, 18 tanks (500 L) were divided into six groups (each had three replicates) including one control group and five individual probiotic strains treatment groups. Similar size 288 mature fish were randomly distributed in six treatments with a stocking density of 16 fish/tank. Treatment was fed with 0 CFU (control), GFB-1 with 1.62 × 10^9^, GFB-2 with 1.43×10^9^, GFB-3 with 1.06×10^9^, GFB-4 with 1.5×10^9^, and GFB-5 with 1.13×10^9^ CFU kg^-1^ feed for an experimental period of 60 days. Fish hand-fed daily at 2.5% of the total biomass, twice daily at 0900 h and 1900 h for a period of 60 days. Feed adjustments were done for each tank every 15 days after sampling. The dough was prepared every 7 days interval. The uneaten feeds were collected during water exchange.

**1b. The experimental unit (e.g., a single animal, litter, or cage of animals).**

**Statement:** To assess the consortium effect, twelve plastic tanks (500L) were divided into four treatments such as T1 (control) (0), T2 (1.35 ×10^9^), T3 (2×1.35 ×10^9^), and T4 (3×1.35 ×10^9^) CFU kg^-1^ feed with three replicates. A total of 192 uniformly sized mature fish were randomly distributed in four treatments and the stocking density was maintained at 16 fish/tank (male: females at 1:3) following a completely randomized design. To determine the individual effect of probiotic strains, 18 tanks (500 L) were divided into six groups (each had three replicates) including one control group and five individual probiotic strains treatment groups. Similar size 288 mature fish were randomly distributed in six treatments with a stocking density of 16 fish/tank.

**Item 2. Sample size**

**2a. Specify the exact number of experimental units allocated to each group, and the total number in each experiment. Also indicate the total number of animals used.**

**Statement:** To assess the consortium effect, twelve plastic tanks (500L) were divided into four treatments such as T1 (control) (0), T2 (1.35 ×10^9^), T3 (2×1.35 ×10^9^), and T4 (3×1.35 ×10^9^) CFU kg^-1^ feed with three replicates. A total of 192 uniformly sized mature fish were randomly distributed in four treatments and the stocking density was maintained at 16 fish/tank (male: females at 1:3) following a completely randomized design. To determine the individual effect of probiotic strains, 18 tanks (500 L) were divided into six groups (each had three replicates) including one control group and five individual probiotic strains treatment groups. Similar size 288 mature fish were randomly distributed in six treatments with a stocking density of 16 fish/tank. Six fishes from each treatment were anesthetized with the clove oil and gradually sacrificed to collect intestine and liver for histological study. At the end of the growth trial experimental period (60 days), a total of 27 fish from each treatment were anesthetized with the clove oil (0.05 mL per 500 mL of water) for hematological analysis. At the end of the experiment period (60 days), a total of 96 mature fish (72 females and 24 males) were selected for the assessment of consortium effect of probiotic and a total of 144 mature fish (108 females and 36 males) mature fish were selected for the assessment of the individual effect of probiotic on reproductive performances. A total of 24 mature fish were selected from each treatment and matured six females and two male broodstocks were selected from each replication. Then eight fish (male: female = 3:1) from each replication were transferred into the individual holding tank and acclimatized for two days. For survival rate measurement, twelve plastic bowls for the consortium of probiotic strains and eighteen plastic bowls for individual probiotic strain each of 10 L capacity were divided into four groups and six groups corresponding to four treatments and six treatments respectively and each of the bowl was stocked with 100 larvae as a stocking rate of 10 larvae/L. At the end of the feeding trial of growth performances, 9 fish from each treatment (3 fish from each replication) by random sampling were examined to assess the effect of probiotic bacteria on gut microbiota.

**2b. Explain how the sample size was decided. Provide details of any a priori sample size calculation, if done.**

**Statement:** Power analysis was performed to check the statistical validity of sample size. The typical power analysis for an ANOVA was performed using G*Power version 3.0.10.

**Item 3. Inclusion and exclusion criteria**

**3a. Describe any criteria used for including or excluding animals (or experimental units) during the experiment, and data points during the analysis. Specify if these criteria were established a priori. If no criteria were set, state this explicitly.**

**Statement:** During the experiment, no experimental animals were included or excluded.

**3b. For each experimental group, report any animals, experimental units, or data points not included in the analysis and explain why. If there were no exclusions, state so.**

**Statement:** For each experimental group, all the data were included in the statistical analysis.

**3c. For each analysis, report the exact value of *n* in each experimental group.**

**Statement:** Value of *n* in each experimental group are given below:

1. Assessment of consortium effects of five probiotic bacterial strains (Figure 2): *n=48.*
2. Assessment of individual effects of five probiotic bacterial strains (Figure 3): *n=48.*
3. Comparative study between assessments of individual effects of five probiotic bacterial strains (Figure 4): *n=48.*
4. Histological analysis of the effects of probiotic bacteria supplementation on villi length of intestine of *B. gonionotus* (Figure 5): *n=6.*
5. Histological analysis of the effects of probiotic supplementation on liver (Figure 6): *n=6.*
6. Assessment of consortium effects of bacterial probiotic strains on hematological parameters (Figure 7): *n=27.*
7. Assessment of individual effects of five isolated probiotic strains on hematological parameters (Figure 8): *n=27.*
8. Comparative study between assessment of individual effects of five probiotic bacterial strains on hematological parameters (Figure 9): *n=27.*
9. Assessment of consortium effects of probiotic bacterial strains on reproductive performances of *B. gonionotus* (Figure 10): *n=24.*
10. Assessment of individual of probiotic bacterial strains on reproductive performances of *B. gonionotus* (Figure 11): *n=24.*
11. Comparative study between assessment of individual effects of five probiotic bacterial strains on reproductive performances of *B. gonionotus* (Figure 12): *n=24.*
12. Assessment of consortium effects of five probiotic bacterial strains and individual effect of five probiotic bacterial strains isolated from the gut of *B. gonionotus* on the culturable autochthonous bacteria and autochthonous lactic acid bacteria levels (Figure 13): *n=9.*
13. The effects of the five gut probiotic bacteria on the digestive enzymes activity (Table2): *n=3.*
14. Antibiotic susceptibility profile of five bacterial probiotic strains isolated from the gut of *B. gonionotus* (Table 3): *n=3.*

**Item 4. Randomisation**

**4a. State whether randomisation was used to allocate experimental units to control and treatment groups. If done, provide the method used to generate the randomisation sequence.**

**Statement:** All the experimental animals were randomly distributed in treatment groups by completely randomized design using a computer based random order generator

**4b. Describe the strategy used to minimise potential confounders such as the order of treatments and measurements, or animal/cage location. If confounders were not controlled, state this explicitly.**

**Statement:** Test time was between twice daily at 0900 h and 1900 h for a period of 60 days, and testing order was randomized daily, with each animal tested at a different time each test day.

**Item 5. Blinding**

**Describe who was aware of the group allocation at the different stages of the experiment (during the allocation, the conduct of the experiment, the outcome assessment, and the data analysis).**

**Statement:** M.A.S. and M.L.R., initiated the research works; M.A.S., conceived the study; M.A.S., M.M.R., T.I., and M.S.A., drafted, edited and interpreted data; M.A.I., F. I., M.A.S., and S.I.P. performed the experiments; M.A.S., and D.C.S., performed statistical analysis; S. I. P., M. A. I., M.M.R., and A.R., performed isolation and molecular identification of bacteria and conducted bioinformatics analysis. Personnel who analyze the data collected from the study are not aware of the treatment applied to any given group.

**Item 6. Outcome measures**

**6a. Clearly define all outcome measures assessed (e.g., cell death, molecular markers, or behavioural changes).**

**Statement:** The following parameters were assessed: growth performances, histological and hematological parameters and reproductive performances of *B. gonionotus*

**6b. For hypothesis-testing studies, specify the primary outcome measure, i.e., the outcome measure that was used to determine the sample size.**

**Statement:** The primary outcome of this study is to the isolation and identification of probiotic bacteria from the gut of *B. gonionotus* using *16S rRNA* gene sequencing.

**Item 7. Statistical methods**

**7a. Provide details of the statistical methods used for each analysis, including software used.**

**Statement:** Data of weight gain, specific growth rate, intestinal villi length, gut microbiota, enzyme activity, gonadsomatic index, ovulation rate, fertilization rate, and hatching success were collected during the study period and statistically analysed using one-way analysis of variance (ANOVA) to test the significant results (P < 0.05) between means and the mean values were separated by LSD (least significance difference) posthoc statistic. Standard deviation (±SD) was calculated to identify the range of means. All statistical analyses were performed with the aid of the computer software Statistix 10.0 version. Power analysis was performed to check the statistical validity of sample size. The typical power analysis for an ANOVA was performed using G*Power version 3.0.10. Cumulative survival of larva were analysed through Kaplan-meier survival analysis in Microsoft Office Excel version 2016. Weight gain data collected were repeated statistically analysed using ANOVA to test significance results (P < 0.05) between means. The standard error (±SE) was calculated to identify the range of means. These statistical analyses were performed with the aid of the computer software SPSS 26.0 version.

**7b. Describe any methods used to assess whether the data met the assumptions of the statistical approach, and what was done if the assumptions were not met.**

**Statement:** The typical power analysis for an ANOVA was performed using G*Power version 3.0.10 to check the statistical validity of data and found all the data statistically valid.

**Item 8. Experimental animals**

**8a. Provide species-appropriate details of the animals used, including species, strain and substrain, sex, age or developmental stage, and, if relevant, weight.**

**Statement:** A total of 480 (average 65.6 ± 0.8g) experimental fish (*B. gonionotus*) were used in this experiment. Sex was identified based on the external morphological characteristics.

**8b. Provide further relevant information on the provenance of animals, health/immune status, genetic modification status, genotype, and any previous procedures.**

**Statement:** Healthy *B. gonionotus* fish were collected from an aquafarm from Trishal, Mymensingh, Bangladesh for probiotic isolation.

**Item 9. Experimental procedures**

**For each experimental group, including controls, describe the procedures in enough detail to allow others to replicate them, including:**

**9a. What was done, how it was done, and what was used.**

**Statement:** Detail protocols are available at protocols.io website ([dx.doi.org/10.17504/protocols.io.bs34ngqw](https://dx.doi.org/10.17504/protocols.io.bs34ngqw)).

**9b. When and how often.**

**Statement:** Fish hand-fed daily at 2.5% of the total biomass, twice daily at 0900 h and 1900 h for a period of 60 days. Feed adjustments were done for each tank every 15 days after sampling. The dough was prepared every 7 days interval. The uneaten feeds were collected during water exchange. Water quality parameters such as, pH, dissolve oxygen, and temperature were routinely measured to maintain the health of fish.

**9c. Where (including detail of any acclimatisation periods).**

**Statement:** The fish were stocked in the circular plastic tanks (500L) with aerators and acclimatized for 15 days in the wet laboratory. Water change was done every 3 days interval and uneaten feeds were collected.

**9d. Why (provide rationale for procedures).**

**Statement:** Not applicable in this research.

**Item 10. Results**

**For each experiment conducted, including independent replications, report:**

**10a. Summary/descriptive statistics for each experimental group, with a measure of variability where applicable (e.g., mean and SD, or median and range).**

**Statement:** Mean ± SD values are presented below:

1. In the case of a consortium application, the weight gains (mean ± SD) of fishes were 6.72 ± 0.68, 24.43 ± 1.12, 16.25 ± 0.84, and 12.56 ± 0.47 g in T1, T2, T3, and T4, respectively after the end of 60 days.
2. The food conversion ratio (FCR) (mean ± SD) in T1, T2, T3, and T4 were 2.17 ± 0.006, 1.94 ± 0.036, 2.03 ± 0.001, and 2.07 ± 0.015, respectively after 60 days.
3. The highest specific growth rate (%/day) (SGR) (mean ± SD) 0.516 ± 0.029 was found in GFB-1 and the lowest SGR 0.303 ±0.026 was found in GFB-4.
4. The highest length (mean ± SD) of intestinal microvilli was 712.317± 23.66 µm in GFB-1 and the lowest length of intestinal microvilli was 446.00±9.85 µm in the untreated control.
5. The highest number (mean ± SD) of lactic acid bacteria were log (5.51±0.23) CFU/g, log (5.41 ± 0.2) CFU/g, log (5.37 ± 0.19) CFU/g, log (5.12±0.12) CFU/g in GFB-1, GFB-2, GFB-3 and consortium (T2), respectively and the number of lactic acid bacteria was log (3.87±0.28) CFU/g in the untreated control.
6. Among the bacterial isolates, significantly (P < 0.05) the highest protease activities (mean ± SD) were demonstrated by GFB-3 (8.60±0.61 µg/ml/hr) followed by GFB-1 (8.43±0.41 µg/ml/hr) and GFB-4 (7.60±0.49 µg/ml/hr) (Table 2). Significantly (P < 0.05) the highest lipase activity was shown by the isolate GFB-1 (1.43±0.03 µmol fatty acid/ml) followed by GFB-2 (1.40±0.03 µmol fatty acid/ml) and significantly (P < 0.05) the lowest lipase activity was recorded for the isolate GFB-4 (1.27±0.06 µmol fatty acid/ml).

**10b. If applicable, the effect size with a confidence interval.**

**Statement:** Power analysis results are listed below:

Test family: F tests

Statistical test: ANOVA: Fixed effects, omnibus, one-way

Type of power analysis: A priori: Computer required sample size – given α, power, and effect size

| **Figure** | **Parameter** | **Value** |
| --- | --- | --- |
| Figure 2 | Effect size f | 10.54 |
|  | α err prob | 0.05 |
|  | Power (1-β err prob) | 0.94 |
|  | Actual power | 1.00 |
| Figure 4 | Effect size f | 4.62 |
|  | α err prob | 0.05 |
|  | Power (1-β err prob) | 0.95 |
|  | Actual power | 1.00 |
| Figure 10A (GSI) | Effect size f | 0.64 |
|  | α err prob | 0.05 |
|  | Power (1-β err prob) | 0.8 |
|  | Actual power | 0.82 |
| Figure 10B (No. of ova) | Effect size f | 10.98 |
|  | α err prob | 0.05 |
|  | Power (1-β err prob) | 0.8 |
|  | Actual power | 1.00 |
| Figure 10C (Fertilization) | Effect size f | 5.88 |
|  | α err prob | 0.05 |
|  | Power (1-β err prob) | 0.8 |
|  | Actual power | 1.00 |
| Figure 10D (Hatching rate) | Effect size f | 5.74 |
|  | α err prob | 0.05 |
|  | Power (1-β err prob) | 0.8 |
|  | Actual power | 1.00 |
| Figure 11A (GSI) | Effect size f | 1.35 |
|  | α err prob | 0.05 |
|  | Power (1-β err prob) | 0.8 |
|  | Actual power | 0.96 |
| Figure 11B (No. of ova) | Effect size f | 9.24 |
|  | α err prob | 0.05 |
|  | Power (1-β err prob) | 0.8 |
|  | Actual power | 1.00 |
| Figure 11C (Fertilization) | Effect size f | 4.03 |
|  | α err prob | 0.05 |
|  | Power (1-β err prob) | 0.8 |
|  | Actual power | 1.00 |
| Figure 11D (Hatching rate) | Effect size f | 4.45 |
|  | α err prob | 0.05 |
|  | Power (1-β err prob) | 0.8 |
|  | Actual power | 1.00 |
| Figure 12A (GSI) | Effect size f | 0.25 |
|  | α err prob | 0.05 |
|  | Power (1-β err prob) | 0.80 |
|  | Actual power | 0.81 |
| Figure 12B (No. of ova) | Effect size f | 8.92 |
|  | α err prob | 0.05 |
|  | Power (1-β err prob) | 0.8 |
|  | Actual power | 1.00 |
| Figure 12C (Fertilization) | Effect size f | 5.12 |
|  | α err prob | 0.05 |
|  | Power (1-β err prob) | 0.8 |
|  | Actual power | 1.00 |
| Figure 12D (Hatching rate) | Effect size f | 5.42 |
|  | α err prob | 0.05 |
|  | Power (1-β err prob) | 0.8 |
|  | Actual power | 1.00 |

**Item 11. Abstract**

**Provide an accurate summary of the research objectives, animal species, strain and sex, key methods, principal findings, and study conclusions.**

**Statement:** This study aimed to isolate and identify probiotic bacteria from the gut of *Barbonymus gonionotus* and evaluate their effects on growth, hematological parameters, and breeding performances of the host. Five probiotic bacteria *viz. Enterococcus xiangfangensis* (GFB-1), *Pseudomonas stutzeri* (GFB-2), *Bacillus subtilis* (GFB-3), *Citrobacter freundii* (GFB-4), and *P. aeruginosa* (GFB-5) were isolated and identified using *16S rRNA* gene sequencing. Application of a consortium of probiotic strains (1-3 ×1.35× 10^9^ CFU kg^-1^) or individual strain such as GFB-1 (1.62 × 10^9^ CFU kg^-1^), GFB-2 (1.43 × 10^9^ CFU kg^-1^), GFB-3 (1.06 × 10^9^ CFU kg^-1^), GFB-4 (1.5 × 10^9^ CFU kg^-1^) or GFB-5 (1.43 × 10^9^ CFU kg^-1^feed) through feed significantly improved growth, histological and hematological parameters and reproductive performances of *B. gonionotus* compared to untreated control. Moreover, the application of these probiotics significantly increased gut lactic acid bacteria and activities of digestive enzymes but did not show any antibiotic resistance nor any cytotoxicity *in vitro*. The highest beneficial effects on treated fishes were recorded by the application of GFB-1, GFB-2, GFB-3, and a consortium of these bacteria (T2). This is the first report of the improvement of growth and health of *B. gonionotus* fishes by its gut bacteria.

**Item 12. Background**

**12a. Include sufficient scientific background to understand the rationale and context for the study, and explain the experimental approach.**

**Statement:** More than 70 percent of antibiotics are used in aquaculture operations wind up in the environment and plasmid carrying resistance genes can be transferred from fish pathogen to humane pathogens. A few studies showed that probiotic bacteria increase immune response and growth performances in fish. Several lines of evidence suggest that application of the native host developed probiotic strains of probiotic bacteria improve the health of host fishes by decreasing the mortality rate. It is reasonable to hypothesize that the probiotic bacteria isolated from the guts of native fishes could be the potential candidates for the promotion of sustainable aquaculture. Host-associated probiotics boosted the growth, immunity, and reproductive performances of the fish. Most authors generally consider growth, immunity, and reproductive performances without focus on the possibility of histological changes caused in the internal organs like the intestine and liver as a result of probiotic use. In this aspect, genetically characterized probiotic bacteria isolated from the gut of native fishes could be used as cheap and eco-friendly agents in enhancing growth performance, hematological parameters, and breeding performances of fishes for promoting sustainable aquaculture.

**12b. Explain how the animal species and model used address the scientific objectives and, where appropriate, the relevance to human biology.**

**Statement:** The 2030 agenda for aquaculture is promoting sustainability by using natural resources as opposed to antibiotic prevention. Surprisingly, world fish production is highly increased to about 171 million tonnes in 2016, where aquaculture represented 47% of the total. In Bangladesh, carp production is about 1.19 million metric tonnes, which is about 32.6% of the total fish production. However, average fish production in aquaculture of Bangladesh is still much lower than many other carp producing countries like China. In this respect, a minor carp, *Barbonymus gonionotus* (Bleeker, 1850), commonly known as silver barb in aquaculture, widely distributed in almost all the countries in the world especially in South East Asian countries for aquaculture. Because of its high popularity, its distribution has been widely extended by human introduction. This species is widely used in polyculture as well as weed control in South East Asia and also important fish species for integrated rice-fish farming. The interest in *B. gonionotus* as a means of biological control of weeds instead of grass carp that destroy the plants. For this reason we use this fish for probiotic isolation and their application.

**Item 13. Objectives**

**Clearly describe the research question, research objectives and, where appropriate, specific hypotheses being tested.**

**Statement:** The objectives of the present study were to (i) isolate and identify probiotic bacteria from the gut of *B. gonionotus* using 16S rRNA gene sequencing; (ii) investigate the effects of individual and consortium application of the identified probiotic bacteria on growth and hematological parameters; and (iii) evaluate their influence on reproductive performances of the host fishes.

**Item 14. Ethical statement**

**Provide the name of the ethical review committee or equivalent that has approved the use of animals in this study and any relevant licence or protocol numbers (if applicable). If ethical approval was not sought or granted, provide a justification.**

**Statement:** All procedures were conducted in accordance with the United Kingdom Animal (Scientific Procedures) Act 1986, approved by Ethical Review Committee (ERC) of the Institute of Biotechnology and Genetic Engineering (IBGE), Bangabandhu Sheikh Mujibur Rahman Agricultural University (BSMRAU), Gazipur-1706, Bangladesh and conducted under the authority of the project Licence BSMRAU/IBGE/002.

**Item 15. Housing and husbandry**

**Provide details of housing and husbandry conditions, including any environmental enrichment.**

**Statement:** The fish were housed in circular plastic tanks (500L) with continuous aeration. Water change was done every 3 days interval and uneaten feeds were collected. Water quality parameters such as, pH, dissolve oxygen, and temperature were routinely measured to maintain the health of fish.

**Item 16. Animal care and monitoring**

**16a. Describe any interventions or steps taken in the experimental protocols to reduce pain, suffering, and distress.**

**Statement:** Prior blood collection, the experimental fish were anesthetized by using 0.05 mL clove oil per 500 mL of water to reduce pain, suffering, and distress.

**16b. Report any expected or unexpected adverse events.**

**Statement:** No adverse events were noticed during the study period.

**16c. Describe the humane endpoints established for the study, the signs that were monitored, and the frequency of monitoring. If the study did not set humane endpoints, state this.**

**Statement:** The research team monitored the experimental fish twice daily. Health was monitored by food intake. Water temperature, dissolved oxygen (DO), and pH of water in each replication under each treatment were recorded every day.

**Item 17. Interpretation/scientific implications**

**17a. Interpret the results, taking into account the study objectives and hypotheses, current theory, and other relevant studies in the literature.**

**Statement:** In summary, our study demonstrated that dietary supplementation of gut probiotic bacteria isolated from *B. gonionotus* promoted growth, hematological parameters, and reproductive performances of the host fishes. The gut bacteria*, E. xiangfangensis* (GFB-1), *P. stutzeri* (GFB-2), and *B. subtilis* (GFB-3) should be considered as promising individual candidates for promoting sustainable aquaculture in Bangladesh. These findings also suggested that a consortium of probiotic bacterial strains such as GFB-1, GFB-2, and GFB-3 supplemented diets could be developed for promoting the growth, hematological parameters, and reproductive performances of silver barb fishes in aquaculture.

**17b. Comment on the study limitations, including potential sources of bias, limitations of the animal model, and imprecision associated with the results.**

**Statement:** Although in this study we collected experimental fish samples from only one source. We did not identified the potential genes from the whole-genome sequence of the studied probiotics in this study. Further studies should be focused on the elucidation of underlying mechanisms of the beneficial effects and safety issues of these gut probiotic bacteria on the host fishes.

**Item 18. Generalisability/translation**

**Comment on whether, and how, the findings of this study are likely to generalise to other species or experimental conditions, including any relevance to human biology (where appropriate).**

**Statement:** Our study demonstrated the effects of five gut probiotic on growth, haematological parameters and reproductive performances of *Barbonymus gonionotus*. Further studies should be focused on the elucidation of underlying mechanisms of the beneficial effects and safety issues of these gut probiotic bacteria on the host fishes. A large-scale field trial of these gut probiotic bacteria is needed before recommending them for practical application in aquaculture.

**Item 19. Protocol registration**

**Provide a statement indicating whether a protocol (including the research question, key design features, and analysis plan) was prepared before the study, and if and where this protocol was registered.**

**Statement:** To maximize the objectivity of the presented research, we preregistered this study with its 2 hypotheses at the Open Science Framework (OSF) (<https://doi.org/10.17605/OSF.IO/E6AXG>).

**Item 20. Data access**

**Provide a statement describing if and where study data are available.**

**Statement:** All the gene sequence data are deposited in the open access repository of National Center for Biotechnology Information (NCBI, <https://www.ncbi.nlm.nih.gov/>). GFB1 (<https://www.ncbi.nlm.nih.gov/nuccore/MK660187.1>),

GFB2 (<https://www.ncbi.nlm.nih.gov/nuccore/MK660190.1>),

GFB3 (<https://www.ncbi.nlm.nih.gov/nuccore/MK660197.1>),

GFB4 (<https://www.ncbi.nlm.nih.gov/nuccore/MK660216.1>), and

GFB5 (<https://www.ncbi.nlm.nih.gov/nuccore/MK660266.1>).

**Item 21. Declaration of interests**

**21a. Declare any potential conflicts of interest, including financial and nonfinancial. If none exist, this should be stated.**

**Statement:** The authors have declared that no competing interests exist.

**21b. List all funding sources (including grant identifier) and the role of the funder(s) in the design, analysis, and reporting of the study.**

**Statement:** This study was financially supported by the Ministry of Science Technology, Bangladesh under the project entitled “Effect of probiotic-based nutrition on hematology and reproduction of silver barb (*Barbonymus gonionotus*, Bleeker, 1850)” and “Isolation, identification and genetic characterization of probiotic bacteria promoting growth and hematology and breeding of silver barb (*Barbonymus gonionotus*, Bleeker, 1850)” to M.A.S to Dept. of Genetics and Fish Breeding. The funders had no role in study design, data collection and analysis, decision to publish, or preparation of the manuscript.
